# Menadione: a platform and a target to valuable compounds synthesis

**DOI:** 10.3762/bjoc.18.43

**Published:** 2022-04-11

**Authors:** Acácio S de Souza, Ruan Carlos B Ribeiro, Dora C S Costa, Fernanda P Pauli, David R Pinho, Matheus G de Moraes, Fernando de C da Silva, Luana da S M Forezi, Vitor F Ferreira

**Affiliations:** 1 Universidade Federal Fluminense, Departamento de Tecnologia Farmacêutica, Faculdade de Farmácia, R. Dr. Mario Vianna, 523, Santa Rosa, CEP 24241-002, Niterói-RJ, Brazil; 2 Department of Chemistry, CICECO – Aveiro Institute of Materials, University of Aveiro, Campus Universitário de Santiago, 3810-193, Aveiro, Portugal; 3 Universidade Federal Fluminense, Instituto de Química, Departamento de Química Orgânica, 24020-150 Niterói, RJ, Brazil

**Keywords:** cancer, chemical reactions, 2-methyl-1,4-naphthoquinone, quinone, synthetic platform, vitamin K

## Abstract

Naphthoquinones are important natural or synthetic compounds belonging to the general class of quinones. Many compounds in this class have become drugs that are on the pharmaceutical market for the treatment of various diseases. A special naphthoquinone derivative is menadione, a synthetic naphthoquinone belonging to the vitamin K group. This compound can be synthesized by different methods and it has a broad range of biological and synthetic applications, which will be highlighted in this review.

## Introduction

**Figure 1:**
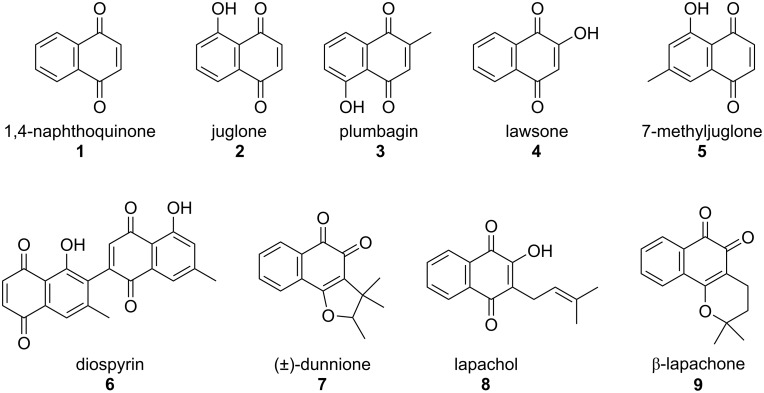
Natural bioactive naphthoquinones.

Naphthoquinones belong to the chemical family of quinones and are widely present in synthetic and natural products (Figure 1). In nature, quinones are biosynthesized as secondary metabolites by various organisms, from simple single-celled microorganisms to more complex beings, such as higher plants and animals [1]. Actually, quinones play important roles in several physiological processes in these organisms, such as photosynthesis [2] and oxidative phosphorylation [3–4], as well as many other metabolic processes [5–7]. Quinones also received considerable attention due to their importance in microbial systems, once several studies have shown that structural variations in microbial quinones have chemotaxonomic significance and can be used in the classification and identification of various microbial species [8]. With their particular and quite interesting chemical properties and bioactivities, naphthoquinones have aroused great interest, mainly in the pharmaceutical field, where they have been widely used in the development of new and more efficient drugs [1,9].

**Figure 2:**
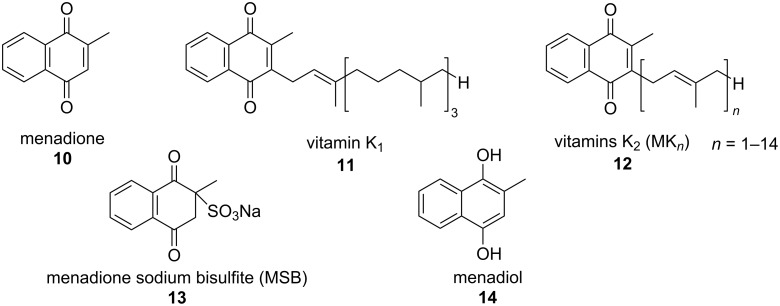
Chemical structures of vitamins K.

The naphthoquinone menadione has attracted a lot of attention. Menadione or 2-methyl-1,4-naphthoquinone (**10**), most known as vitamin K_3_, is a naphthoquinone derivative exclusively synthetic, not found in nature, used as an important precursor to synthesize vitamins K_1_ and K_2_, being classified as a provitamin (Figure 2) [10]. Vitamins K, obtained through food, play an important role in maintaining animals’ physiology, by acting on blood clotting and regulating bone calcification [10]. In animals, menadione can be converted in vitamin K_2_ in the intestinal tract, by intestinal microbiota [10]. In humans, the menadione–vitamin K_2_ conversion occurs after its alkylation in the liver [11]. Moreover, in adult humans, vitamin K_1_ can be converted into vitamin K_2_, a process that requires menadione as intermediate [12]. Menadione sodium bisulfite complex (MSB, **13**) [13] and menadiol (vitamin K_4_, **14**) [14], in turn, are two water-soluble derivatives converted in the body, to menadione. The MSB favors the formation of prothrombin and speeds up blood coagulation, improving its antihemorrhagic activity when compared to the natural vitamins K [13].

The action of menadione in live organisms is not restricted to its use as a biosynthetic precursor to vitamins K_1_ and K_2_, a variety of studies has shown a wide range of biological activities of menadione, such as anticancer [15–22], antibacterial [23–26], antifungal [27–28], antimalarial [29–32], antichagasic [33], and anthelmintic [34] effects. In these cases, the redox cycle of menadione, followed by reactive oxygen species (ROS) generation, resulted from the interactions between nucleophilic biomolecules and 1,4-naphthoquinonic nucleus of menadione and its derivatives.

**Figure 3:**
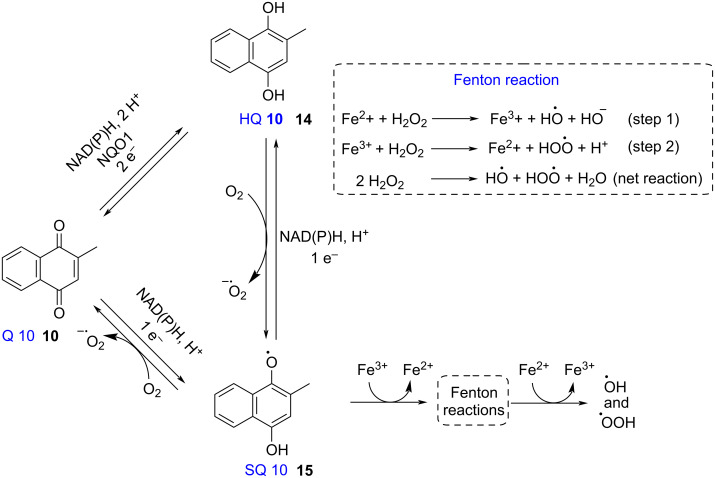
Redox cycle of menadione.

The presence of an α,β-unsaturated diketone in the quinone structures allows them to accept electrons through reduction processes, followed by oxidation, thus establishing a redox cycle. The main characteristics of the quinones (Q) redox cycle, comprises the one-electron reduction to generate a semiquinone intermediate (SQ) and the two-electron reduction leading to hydroquinone (HQ), in NAD(P)H oxidase-dependent processes [35–38]. In the presence of oxygen, the reduced species is oxidized back to the quinone, thus completing the cycle. In case of naphthoquinones such as menadione, the quinone–semiquinone or quinone–hydroquinone interconversion generates reactive oxygen species (ROS), such as superoxide anion (O_2_^•−^), hydrogen peroxide (H_2_O_2_), hydroxyl radical (^•^OH), and hydroperoxyl radical (^•^OOH) (Figure 3) [35]. Additionally, the menadione semiquinone radical can participate in another redox cycle, such as, the Fenton reaction, also resulting in the production of hydroxyl and hydroperoxyl radicals (Figure 3) [39–41].

The quinone–hydroquinone and quinone–semiquinone interconversions, with ROS generation, are responsible for the wide range of biological activities of menadione and its derivatives. An excess of ROS in the intracellular environment can cause harmful effects on structures such as nucleic acids (DNA and RNA), enzymes, structural proteins, and cell membrane lipids. These effects can lead to dysfunctions that activate the apoptosis process resulting in cell death [42–46].

In addition to participating in redox cycles as biomolecules in various biochemical processes, the 1,4-naphthoquinone structure of the menadione core is also easily recognized and incorporated by different live organisms, as is evidenced by its presence in the structure of several natural naphthoquinones. The ability to participate in biochemical processes by interacting with biomolecules through redox cycles makes menadione a very interesting structural model for the development of new bioactive compounds. Considering the great potential of menadione, mainly in biological applications, in this review several applications involving its synthesis and its use as a versatile synthetic platform will be discussed.

## Review

### Preparation methods of menadione

Among the most common methods for the preparation of menadione (**10**), we can find, for example, the oxidation of 2-methylnaphthalene or 2-methylnaphthol. Other less frequent but equally efficient approaches to synthesize menadione include the demethylation of 2-methyl-1,4-dimethoxynaphthalene, the construction of the naphthoquinone ring, the methylation of 1,4-naphthoquinone, and the electrochemical synthesis from 2-methyl-1,4-dihydroxynaphthalene. The works discussed in this section are grouped according to the synthetic approach that was employed to prepare menadione.

#### Oxidation of 2-methylnaphthalene

Menadione synthesis through the oxidation of 2-methylnaphthalene (**16**) includes the use of oxygen-rich oxidants using various reaction conditions and a broad range of well-succeeding methodologies has been reported. A summary is presented in Table 1 and will be discussed in this section.

**Table 1:** Different approaches of 2-methylnaphthalene oxidation to menadione.

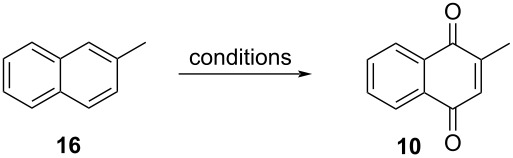				

Entry	Conditions	Catalyst	Yield (%)	Ref.

1	CrO_3_, H_2_O, AcOH, 85–90 °C, 1 h	–	38–42	[47]
2	Na_2_Cr_2_O_7_·H_2_O, H_2_SO_4_, CCl_4_, 80 to 0 °C, 15 min	–	48–62	[48]
3	H_5_IO_6_, CH_3_CN, 5 °C, 1 h	CrO_3_	61	[49]
4	H_2_O_2_ (60%), AcOH, 50 °C, 8 h	palladium(II)-resin	50–60	[50]
5	H_2_O_2_ (85%), Ac_2_O, AcOH, 40 °C, N_2_, 4 h	CH_3_ReO_3_	46	[51]
6	H_2_O_2_ (30%), AcOH, 100 °C, 3 h	–	86	[52]
7	H_2_O_2_ (0.2 M), AcOH, rt, 24 h	iron(III) salts	13	[53]
8	H_2_O_2_ (30%), AcOH, 60 °C, 3 h	MnPc	60	[54]
9	H_2_O_2_ (30%), TAA, rt, 1.5 h	FeCl_3_·H_2_O/H_2_Pydic/benzylamine	44	[55]
10	H_2_O_2_, AcOH, 100 °C, 6 h	SeMCM-41	99^a^	[56]
11	H_2_O_2_ (30%), AcOH, H_2_SO_4_, 60–80 °C, 1 h	[(DIPAPTES)PdCl_2_] or [SiO_2_(DIPAPES)PdCl_2_]	52 or 59	[57]
12	H_2_O_2_ (35%), CH_3_CN, AcOH, reflux, 8 h	GO@CHONHRN(CH_2_PPh_2_)_2_PdCl_2_	99^a^	[58]
13	H_2_O_2_ (35%), CH_3_CN, AcOH, H_2_SO_4_, reflux, 12 h	L1-iron(III)	79^a^	[59]
14	H_2_O_2_ (35%), CH_3_CN, H_2_O, 60 °C, 20 min	H[Cu^II^(ttb)(H_2_O)_3_]_2_[Cu^II^(ttb)Cl]_2_ [PW_12_O_40_]·4H_2_O	78^a^	[60]
15	CH_3_COOOH, AcOH, 80 °C, 2 h	Au/HPS	72	[61]
16	O_3_, AcOH, 160 °C	chromium(III) and manganese(II) salts	70	[62]

^a^Conversion.

In a pioneering study, Fieser reported the use of chromium(IV) oxide in glacial acetic acid for the oxidation of 2-methylnaphthalene (**16**) and obtained menadione (**10**) in 38–42% yield (Table 1, entry 1) [47]. A similar process was developed by Li and Elliot, who used sodium dichromate as oxidizing agent in the presence of sulfuric acid, instead of acetic acid, to obtain compound **10** in 62% yield within a shorter reaction time (Table 1, entry 2) [48]. The methodology using sodium dichromate and sulfuric acid was adapted to the industrial scale production of vitamin K_3_ (**10**). However, this process is not ecofriendly, once, in this reaction, 18 kg of inorganic salts were obtained as a byproduct per kg of product and it was necessary to treat the wastewater containing chromium. In this context, alternative approaches with a broad range of catalysts and oxidizing agents were studied [63]. Yamazaki reported the use of 10 mol % of chromium(VI) oxide and orthoperiodic acid, as a terminal oxidant, to obtain menadione (**10**) in 61% yield (Table 1, entry 3) [49].

Alternatives to chromium(VI) compounds to oxidize 2-methylnaphthalene (**16**) to menadione (**10**) have also been evaluated and one of the most studied and used oxidants has been H_2_O_2_. Yamaguchi and co-workers described the oxidation of **16** with aqueous H_2_O_2_ in the presence of a palladium(II)-polystyrene sulfonic acid resin (Table 1, entry 4) [50]. According to the authors, in the absence of the catalysts, the oxidation took place slowly with 7.8% yield, meanwhile, Pd-catalysis improved the yield to 50–60% under otherwise identical conditions [50]. The approach described by the Adam’s group used H_2_O_2_ (85%) and methyltrioxorhenium(VII) (MTO) as the catalyst (Table 1, entry 5) [51,64]. Without the catalyst, the reaction yield was only 10%, but after the addition of MTO and acetic anhydride, the yield increased to 46% (Table 1, entry 5) [51]. The authors suggested two simultaneous reaction pathways: a direct oxidation by rhenium bisperoxo complex and an MTO-catalyzed in situ generation of peroxyacetic acid as oxidant from acetic anhydride [64]. Later in 2002, Narayanan and co-workers reported the oxidation of **16** with H_2_O_2_ (30%) in acetic acid at 100 °C without catalyst (Table 1, entry 6) [52]. The authors obtained the desired product **10** in 86% yield and 95% conversion. This approach could represent a cheap and more ecofriendly method for the synthesis of **10**, because it avoids mineral acid and chromium salts [52]. Sobkowiak and co-workers reported the use of iron(III) as a catalyst to activate H_2_O_2_ for the oxidation of **16** in glacial acetic acid (Table 1, entry 7) [53]. However, the oxidation process was not selective, and traces of 6-methyl-1,4-naphthoquinone were also identified. The reaction yields are not dependent on the type of the iron(III) salt used (perchlorate or acetate), except for iron(III) chloride, which exclusively leads to 1-chloro-2-methylnaphthalene as product [53].

Xiao and co-workers reported a manganese(II) naphthenate (MnPc)-catalyzed oxidation of **16** to furnish **10** in 60% yield with 75.6% conversion and 80% selectivity (Table 1, entry 8) [54]. The MnPc catalyst improves the stability of H_2_O_2_ and thus promotes a selective oxidation [54]. The approach developed by Beller’s group, which applied a three component catalyst system consisting of iron(III) chloride, pyridine-2,6-dicarboxylic acid (H_2_Pydic), and benzylamine (1:1:2.2) for the oxidation of **16** with H_2_O_2_, in *tert*-amyl alcohol (TAA), allowed to obtain **10** in 44% yield (Table 1, entry 9) [55]. Subsequently, Kulkarni’s group evaluated the catalytic activity of selenium mesoporous molecular sieves (SeMCM-41) in the oxidation of **16** (Table 1, entry 10). The approach was performed using H_2_O_2_ as oxidant in acetic acid over SeMCM-41 (Si/Se = 30) at 100 °C [56]. In this case, a conversion of 99% was achieved and menadione (**10**) was obtained with 68% selectivity. According to the authors, the reaction mechanism involves the formation of an active selenium peroxo species. Additionally, they mentioned that the catalyst was easily separated from the reaction mixture by simple filtration [56].

Serindağ and co-workers disclosed the bidentate tertiary aminomethylphosphine complexes of Ru(II), Pd(II), and Co(II) with *N*,*N*-bis(diphenylphosphinomethyl)aminopropyltrietoxysilane (DIPAPTES) and the best yields were obtained using [(DIPAPTES)PdCl_2_] complex and silica supported [SiO_2_(DIPAPES)PdCl_2_] complex, in 52% and 59% yields, respectively (Table 1, entry 11). In both cases, the formation of product **10** was observed with conversions of up to 90% [57]. In another approach, Uruş and co-workers used graphene oxide (GO)-supported bis(diphenylphosphinomethyl)amino GO@CHONHRN(CH_2_PPh_2_)_2_MX_2_ (M:Pd(II) and Pt(II))-type complexes as heterogeneous nanocatalysts, with Pd(II) complexes showing the best catalytic activities with high selectivity compared to the Pt(II) complex, leading to 95–99% conversion and 60–65% selectivity (Table 1, entry 12) [58].

Sönmez and co-workers applied mononuclear complexes of ruthenium(III), chromium(III), and iron(III) with Schiff base ligands as catalyst for the oxidation of 2-methylnaphthalene (**16**) with H_2_O_2_ [59]. The complex L1-Fe(III) (L1 = (2-((2-(2-((2-((2-hydroxyphenylimino)methyl)phenoxy)methyl)benzyloxy)benzylidene)amino)phenol) showed the best catalytic activity with 58.54% selectivity and 79.11% conversion (Table 1, entry 13) [59]. Chang’s group reported the use of 3D crystalline polyoxometalate-based coordination polymers (POMCPs) as heterogeneous catalysts, with H_2_O_2_, to synthesize **10** and the best result was obtained using H[Cu^II^(ttb)(H_2_O)_3_]_2_[Cu^II^(ttb)Cl]_2_[PW_12_O_40_]·4H_2_O (Httb = 1-(tetrazol-5-yl)-4-(triazol-1-yl)benzene) as the catalyst (Table 1, entry 14) [60].

Other less common oxidizing agents have also been used for the oxidation of 2-methylnaphthalene (**16**) to produce menadione (**10**). Sulman and co-workers, for instance, achieved the oxidation of **10** using peracetic acid as oxidant in the presence of gold nanoparticles deposited on hypercrosslinked polystyrene (Au/HPS) (Table 1, entry 15). The best result was obtained using 1% Au/HPS in glacial acetic acid, which led to 96% conversion and 75% selectivity [61]. Another interesting example of oxidation process was reported by Mamchur and Galstyan, who used ozone as oxidizing agent in the presence of a mixture of chromium(III) and manganese(II) salts to furnish product **10** in 70% yield (Table 1, entry 16) [62]. The authors proposed that the oxidation involves an initial ozonation of the transition metal salts, which then oxidized substrate **16** to the desired product **10**.

#### Oxidation of 2-methylnaphthol

Menadione synthesis was also achieved by oxidation of 2-methylnaphthol (**17**). The main advantage of using substrate **17**, compared to 2-methylnaphthalene, is to avoid the formation of byproducts such as 6-methyl-1,4-naphthoquinone [65]. The conditions for the oxidation of 2-methylnaphthol (**17**) to menadione (**10**) are quite similar to those employed for the oxidation of 2-methylnaphthalene (**16**) using H_2_O_2_, molecular oxygen, and *tert*-butyl hydroperoxide as oxidizing agents.

Similar to the oxidation of compound **16**, it is possible to oxidize 2-methylnaphthol (**17**) with H_2_O_2_ to produce menadione (**10**), as was demonstrated by Minisci and co-workers [66]. In this work, the oxidation of **17** with 60% aqueous hydrogen peroxide, using bromine and sulfuric acid as catalysts, provided menadione in 90% yield (Table 2, entry 1) [66]. According to the proposed mechanism, the first step involves the electrophilic bromination of the corresponding phenol, followed by hydrolysis promoted by H_2_O_2_ [66].

**Table 2:** Approaches for the oxidation of 2-methylnaphthol to menadione (**10**).

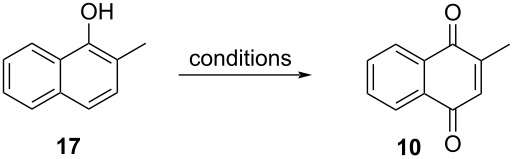				

Entry	Conditions	Catalyst	Yield (%)	Ref.

1	H_2_O_2_ (60%), MeOH, reflux, 20 min	Br_2_, H_2_SO_4_	90	[66]
2	H_2_O_2_ (30%), MeCN, 80 °C, 30 min	Ti-MMM-2	78	[65]
3	H_2_O_2_ (30%), MeCN, reflux, 40 min	TiSBA-15	93	[67]
4	H_2_O_2_, acetone, 70 °C, 2 h	Nb_2_O_2_-SiO_2_	60	[68]
5	H_2_O_2_, MeCN, 75 °C, 45 min	NbSBA-15(2.2pH)	97	[69]
6	H_2_O_2_/CH_3_COOH, CO_2_ (150 bar), 50 °C, 2 h	Au (5%)/HPS	89	[70]
7	H_2_O_2_ (30%), TAA, 0 °C, 1 h	FeCl_3_·H_2_O/H_2_Pydic/benzylamine	55	[55]
8	CO_2_, benzene/H_2_O, 50 °C, 25 min	HPA-*n*	81^a^	[71]
9	O_2_ (3 atm), toluene, 80 °C, 6 h	Au/TiO_2_	57	[72]
10	O_2_ (3 atm), toluene, 80 °C, 6 h	Au/C-2	49	[72]
11	O_2_ (3 atm), toluene, 80 °C, 8 h	–	80	[73]
12	*t*-BuOOH, DCM, 80 °C, 1 h	FePcS-SiO_2_	55	[74]

^a^Conversion.

Variations in the methods of 2-methylnaphthol (**17**) oxidation to menadione (**10**) with H_2_O_2_ were made by changing the catalytic systems in order to increase the yield and selectivity. These include the catalysis by Ti-based [65,67], Nb-based [68–69], and Au-based [70] heterogeneous systems. In addition to being more efficient, given the obtained atom economy, the reactions that use these catalytic heterogeneous systems are also presented as environmentally friendly. They are cleaner, either because of the low generation of waste or the use of environmentally friendly conditions, and they also allow recycling catalysts without losing efficiency [67–68,70].

Kholdeeva and co-workers reported the use of 30% aqueous H_2_O_2_ as oxidant and hydrothermally stable mesoporous mesophase titanium silicates (Ti-MMM-2) as catalyst group, producing **10** in 78% yield at 100% substrate conversion (Table 2, entry 2) [65]. During the studies it was observed that crucial factors affected the product yield, such as substrate concentration, H_2_O_2_/substrate molar ratio, solvent nature, reaction temperature, and mesoporous size [65]. Selvaraj and co-workers reported the liquid-phase oxidation of **17** using Ti-containing mesoporous silica catalysts, TiSBA-15 (Table 2, entry 3) [67]. According to the authors, the best result was achieved with TiSBA-15 (*n*_Si_/*n*_Ti_ = 6) catalysis and H_2_O_2_, exhibiting 93% selectivity to menadione (**10**). In addition, catalyst recycling experiments showed the TiSBA-15(6) had higher catalytic stability in the liquid-phase oxidation as compared to other titanium-containing mesoporous catalysts [67].

Cavani and co-workers reported a heterogenous catalyst system for the oxidation of 2-methylnaphthol (**17**) using 35% aqueous H_2_O_2_ and niobium oxide dispersed in silica (Nb_2_O_2_-SiO_2_) as catalyst (Table 2, entry 4) to obtain menadione (**10**) in 60% yield [68]. Another approach using niobium was developed by the Selvaraj group, which used mesoporous NbSBA-15 catalysts in the liquid-phase oxidation of **17** (Table 2, entry 5) [69]. Different NbSBA-15 catalysts were evaluated and with the optimized reaction conditions, menadione (**10**) was synthetized in 100% conversion and 97.3% selectivity using NbSBA-15(2.2 pH) and H_2_O_2_ [69].

Sulman and co-workers, for instance, reported the synthesis of menadione (**10**) using supercritical (SC) carbon dioxide as green solvent [70]. The authors studied the oxidation using three metal-supported hypercrosslinked polystyrene (HPS) catalysts, which were Au (5%)/HPS, Pd (5%)/HPS and Pt (5%)/HPS, in SC CO_2_ medium. The best conversion (89%) was obtained with Au (5%)/HPS, using CO_2_ (150 bar), and a mixture of H_2_O_2_ and acetic acid as oxidant. Additionally, the selectivity of this process was 99% (Table 2, entry 6).

Beller et al. developed another approach using H_2_O_2_ as oxidizing agent in combination with a three component catalyst system consisting of FeCl_3_·6H_2_O, pyridine-2,6-dicarboxylic acid (H_2_Pydic), and different benzylamines (1:1:2.2) (Table 2, entry 7). The reaction was carried out in *tert*-amyl alcohol (TAA), which led to product **10** in 55% yield and 99% conversion of **17** [55]. In addition to hydrogen peroxide, other oxidizing agents can be used in the synthesis of menadione (**10**) from **17** and include heteropoly acids [71], molecular oxygen [72–73], and organic peroxides [74].

Matveev and co-workers studied phosphomolybdovanadium heteropoly acids of Keggin-type with the general structure H_3+_*_n_*PMo_12-_*_n_*V*_n_*O_40_ (HPA-*n*) and their acidic salts as reversibly acting oxidants to convert **17** to **10** (Table 2, entry 8) [71]. The reaction was carried out in a two-phase solvent system under CO_2_ atmosphere and the best selectivity (89%) was achieved using H_5_PMo_10_V_2_O_40_. In the proposed mechanism, first the HPA-*n* was reduced by **17**, followed by product isolation, and regeneration of HPA-*n* by dioxygen.

The Kholdeeva group also reported the oxidation of 2-methylnaphthol (**17**) using molecular oxygen in the presence of gold nanoparticles as catalyst and the best yield of menadione (**10**) was obtained using 1.5% Au/TiO_2_ as catalyst (57%, Table 2, entry 9), while the best conversion of **17** was furnished using 1% Au/C-2 catalyst (94%, Table 2, entry 10) [72]. In 2011, the same group patented a 2-methylnaphthol (**17**) oxidation approach using molecular oxygen in absence of catalyst under mild reaction conditions (Table 2, entry 11) [73]. The authors reported an oxidation study for this approach involving three alternative reaction mechanisms: free radical autoxidation, cation radical autoxidation, and thermal intersystem crossing (ISC), using ^18^O_2_ labeling, spin-trapping, spectroscopic, mass spectrometric, kinetic, and computational techniques. After several experiments, the obtained results have demonstrated that the 2-methylnaphthol (**17**) oxidation occurs via a thermal ISC (spin inversion) [73]. Additionally, a zwitterionic intermediate formed in the rate-limiting step contributes significantly to the O_2_-based selective oxidation. However, this oxidation mechanism could be modified by the addition of initiators or bases and the predominant path depends mainly on the dioxygen pressure and the solvent nature [73].

Another approach was reported by Zalomaeva and co-workers, which used an iron tetrasulfophthalocyanine (FePcS) supported catalyst (FePcS-SiO_2_) in combination with the oxidizing agent *tert*-butyl hydroperoxide for the oxidation of 2-methylnaphthol (**17**) [74]. Interestingly, the oxidation was efficient with only 0.25 mol % of the catalyst providing 55% selectivity and approximately 50% yield. Indeed, the best product yield (approximately 55%) was achieved at a 95% conversion and 55% selectivity, using dichloromethane and *tert*-butyl hydroperoxide at 80 °C (Table 2, entry 12).

#### Methylation of 1,4-naphthoquinone

Another route to prepare menadione (**10**) involves the methylation of 1,4-naphthoquinone. Because of their electron-deficient character, quinones are highly reactive with nucleophilic radicals [75]. The most useful alkylation approach is the Kochi–Anderson method [76] (or also known as Jacobsen–Torssell method [77–78]), via oxidative decarboxylation, where the quinone reacts with a carboxylic acid in the presence of silver(I) nitrate and ammonium or potassium peroxydisulfate. Nucleophilic free radicals are generated from the carboxylic acid through decarboxylation mediated by [Ag^+^]-peroxydisulfate, followed by their coupling with the quinone, providing the respective alkylated product.

**Scheme 1:**
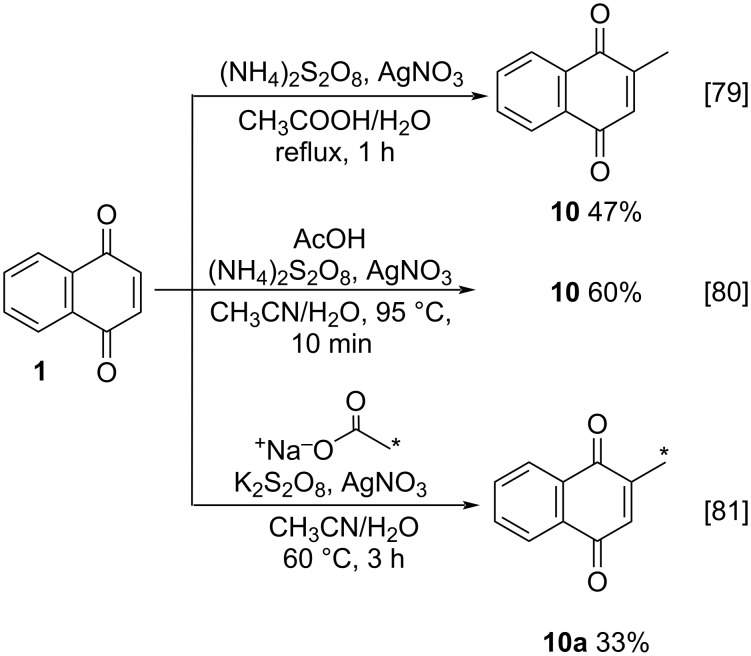
Selected approaches for menadione synthesis using silver(I) as a catalyst.

One of the first practical applications of this methodology to produce menadione (**10**) was described by Ashnagar and co-workers [79]. 1,4-Naphthoquinone (**1**) was treated with acetic acid in the presence of ammonium persulfate, as oxidizing agent, and silver(I) catalysis for only 1 hour, furnishing menadione (**10**) in 47% yield (Scheme 1). After this pioneering work, some adaptations were reported. As an example, Liu and co-workers synthetized **10** using a much simpler way (Scheme 1) [80]. These authors reported the methylation and alkylation of 1,4-naphthoquinone (**1**) in the presence of (NH_4_)_2_S_2_O_8_ and AgNO_3_ as catalyst to obtain **10** in 60% yield. Recently, Onuki and co-workers conducted dimerization reactions of **10**, exploring an interesting artifice to track the dimerization reaction path: they synthesized 2-(methyl-^13^C)-1,4-naphthoquinone (**10**) (Scheme 1) [81]. For that, sodium acetate-2-^13^C was used as the source of the methyl radical, generated by its treatment with K_2_S_2_O_8_ and AgNO_3_. After 3 hours at 60 °C, the ^13^C-labelled menadione was obtained in 33% yield.

**Scheme 2:**
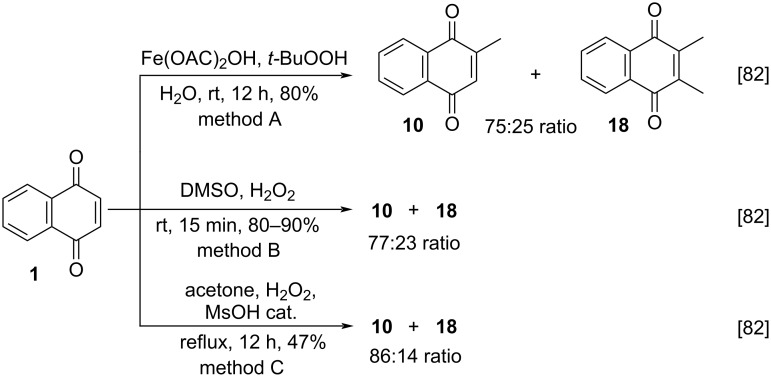
Methylation approaches for the preparation of menadione from 1,4-naphthoquinone using *tert*-butyl hydroperoxide or hydrogen peroxide.

In 1991, Coppa and co-workers reported the homolytic methylation of 1,4-naphthoquinone (**1**) using simple sources of methyl radicals [82]. In the methylation reaction using *tert*-butyl hydroperoxide and Fe(OAc)_2_OH, menadione (**10**) and 2,3-dimethyl-1,4-naphthoquinone (**18**) were obtained in 80% yield with a 75:25 ratio, respectively (Scheme 2, method A). In the methylation reaction using methyl radicals generated by the redox decomposition of H_2_O_2_ in DMSO solution, compounds **10** and **18** were obtained in an overall yield of 80–90% with a 77:23 ratio, respectively (Scheme 2, method B). Finally, the use of H_2_O_2_ thermal decomposition in acetone with catalytic methanesulfonic acid, led to compounds **10** and **18** in 47% yield with 86:14 ratio (Scheme 2, method C). In all cases, the monomethylation was not selective and even at partial conversions of naphthoquinone, significant amounts of dimethyl derivatives were formed. The authors explained the unfavorable steric and polar effects of the methyl group in the quinone ring were probably very low or they were balanced by the favorable enthalpic effects.

**Scheme 3:**
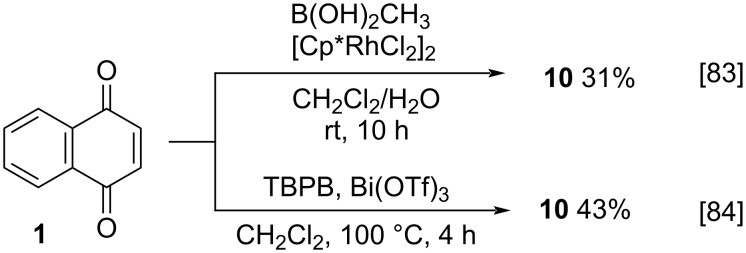
Methylation approach of 1,4-naphthoquinone using i) rhodium complexes/methylboronic acid and ii) bismuth(III) triflate and *tert*-butyl hydroperoxide.

Another interesting work was reported by Wang and co-workers, who studied rhodium complexes as catalysts for the arylation and alkylation of benzo- and naphthoquinones [83]. They synthesized menadione (**10**) (Scheme 3) by reacting 1,4-naphthoquinone (**1**) with methylboronic acid in the presence of [Cp*RhCl_2_]_2_ as catalyst for 10 h, in 31% isolated yield. Later in 2019, Yang and co-workers performed a bismuth catalyst system study for the methylation and alkylation of quinone derivatives [84]. Furthermore, they also evaluated the methylation without catalysts and with the use of lanthanum(II) and copper(II) salts as additive. However, the best results were achieved with bismuth(III) triflate. The use of *tert*-butyl hydroperoxide in the presence of bismuth(III) triflate for the methylation of 1,4-naphthoquinone (**1**) provided **10** in 43% yield, in already optimized conditions (Scheme 3).

#### Demethylation of 2-methyl-1,4-dimethoxynaphthalene

The oxidative demethylation of 1,4-dimethoxyarenes is another valid synthetic approach to achieve 1,4-quinones, with the oxidative demethylation of 2-methyl-1,4-dimethoxynaphthalene (**19**) can be used to synthesize menadione (**10**). The oxidizing agents most commonly used in oxidative demethylation are cerium(IV) ammonium nitrate (CAN), and silver(II) oxide. However, the application of these oxidants is limited, as the synthesis of quinones requires milder reaction conditions. With the purpose of obtaining milder conditions applicable to the synthesis of more complex quinones, including menadione (**10**), some oxidative demethylation methods have been developed based on other oxidizing agents, such as cobalt(III) fluoride [85], phenyliodine(III) bis(trifluoroacetate) (PIFA) [86] and *tert*-butyl hydroperoxide [87] (Table 3).

**Table 3:** Different approaches of 2-methyl-1,4-dimethoxynaphthalene oxidation to menadione.

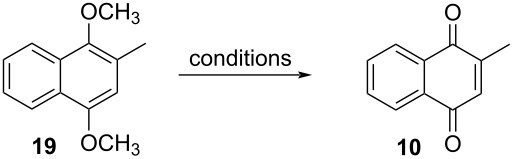				

Entry	Conditions	Catalyst	Yield (%)	Ref.

1	CoF_3_, dioxane, 25 °C, 6 h	–	92	[85]
2	PIFA, MeOH/H_2_O, rt, 30 min	–	92	[86]
3	*t*-BuOOH, *t*-BuOH, 80 °C, 12 h	poly(bis-1,2-phenyl)diselenide	90	[87]

In 1999, Tomatsu and co-workers performed the synthesis of menadione (**10**) through demethylation of 2-methyl-1,4-dimethoxynaphthalene (**19**), using cobalt(III) fluoride as oxidizing agent (Table 3, entry 1) [85]. The obtained results showed that the cobalt(III) fluoride catalyst was comparable with other oxidizing agents already well-established for this synthesis, like silver(II) oxide and ammonium cerium(IV) nitrate. Cobalt(III) fluoride proved to be a good oxidizing agent for the synthesis of menadione (**10**). This approach furnished **10** in 92% yield, although the reaction required a longer reaction time compared to the few minutes using AgO and CAN.

Another problem associated with the use of oxidizing metallic agents is the generation of metallic residues that can be toxic to the environment or act as contaminants of the desired products, such as pharmaceuticals. This problem has led to the search for clean and environmentally friendly methods that reduce or do not generate metallic residues. In 2001, Tohma and co-workers published an alternative and sustainable methodology, using phenyliodine(III) bis(trifluoroacetate) (PIFA) as an oxidizing agent of the demethylation reaction [86]. The hypervalent iodine(III) proved to be a good oxidizing agent in the formation of **10** (92% yield) (Table 3, entry 2). According to the authors, this is a good synthetic path, since PIFA has a low toxicity and it is easily accessible. Subsequently, Wójtowicz and co-workers studied a series of experiments in order to test the oxidative action of *tert*-butyl hydroperoxide and the role of organoselenes as catalysts in the demethylation reaction of hydroquinone ethers [87]. The combination of *tert*-butyl hydroperoxide and poly(bis-1,2-phenyl)diselenide was the one that showed the best result: 90% yield after 12 h reaction and recrystallization (Table 3, entry 3).

#### Construction of the naphthoquinone ring

In addition to the oxidation of naphthalene derivatives, the construction of the naphthoquinone ring is also a viable synthetic method to produce menadione. This is a very efficient strategy with great synthetic value, however, it is less used when compared to other methodologies, as it involves more steps and sometimes more complex reactions.

**Scheme 4:**
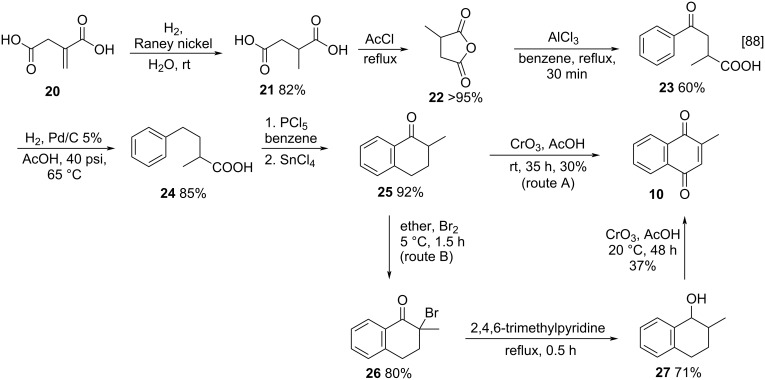
Synthesis of menadione (**10**) from itaconic acid.

One of the pioneering methods for naphthoquinone ring construction was reported by Horii and co-workers who performed the synthesis of menadione (**10**) from itaconic acid, via 2-methyl-1-tetralone [88]. Two synthetic routes were performed (Scheme 4); route A proceeds via the oxidation of 2-methyl-1-tetralone (**25**) using chromium(IV) oxide and route B starts with C-2 bromination of **25**, giving the intermediate **26**, which was reduced to **27**, and oxidized in the presence of chromium(IV) oxide leading to **10** with 37% yield. Although path B was the more complicated to perform, it was the higher yielding route.

**Scheme 5:**
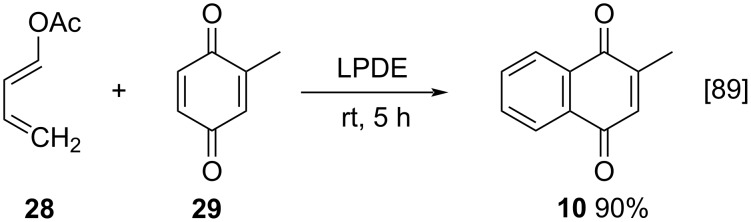
Menadione synthesis via Diels–Alder reaction.

In 2002, an interesting methodology for menadione synthesis was reported by Kacan and Karabulut (Scheme 5). The authors studied a Diels–Alder reaction, using LiClO_4_-diethyl ether (LPDE) as a catalyst, 1-ketoxy-1,3-butadiene **28** as a diene and 2-methyl-1,4-benzoquinone (**29**) as dienophile. By this method, menadione was obtained in 90% yield in 5 hours [89].

**Scheme 6:**
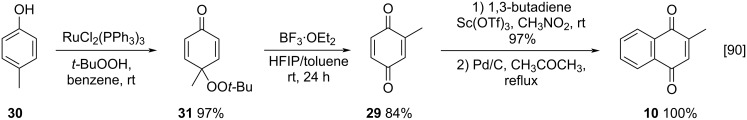
Synthesis of menadione (**10**) using *p*-cresol as a synthetic precursor.

Another route was developed by Murahashi and co-workers that used *p*-cresol as synthetic precursor [90]. First, *p*-cresol, in the presence of *tert*-butyl hydroperoxide, was oxidized to 4-methyl-4-*tert*-butyldioxycyclohexadienone using tris(triphenylphosphine)ruthenium(II) dichloride as catalyst. Then, a BF_3_·OEt_2_-catalzyed migration of the methyl group to the C-2 position and removal of the *tert*-butoxy group in a 1,1,1,3,3,3-hexafluoroisopropanol (HFIP)/toluene mixture afforded 2-methyl-1,4-benzoquinone (**29**). Finally, a Diels–Alder reaction was performed with 1,3-butadiene, followed by dehydrogenation gave menadione (**10**). This proved to be a good synthetic route, leading to menadione in approximately 80% overall yield (Scheme 6).

**Scheme 7:**
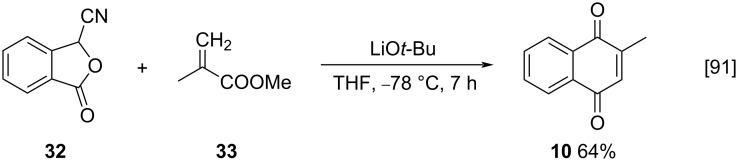
Synthesis of menadione (**10**) via demethoxycarbonylating annulation of methyl methacrylate.

Another interesting synthetic approach was reported by Mal and co-workers, who synthetized menadione (**10**) via a demethoxycarbonylating annulation of methyl methacrylate (**33**) with 3-cyanophthalide (**32**), in the presence of lithium *tert*-butoxide as catalyst (64% yield) (Scheme 7) [91].

**Scheme 8:**
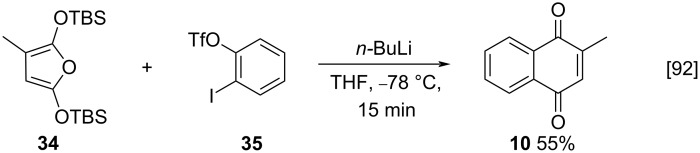
Furan **34** used as a diene in a Diels–Alder reaction for the synthesis of menadione (**10**).

Recently, Dissanayake and co-workers tested the stability of furans to be used as a diene in Diels–Alder reactions for the synthesis of *p*-benzoquinones and *p*-hydroquinones or the synthesis of menadione (**10**). The furan derivative **34** was used as a diene and 2-iodophenyltrifluoromethanesulfonate (**35**) as a dienophile, in the presence of *n*-butyllithium, forming **10** in 55% yield (Scheme 8) [92].

**Scheme 9:**
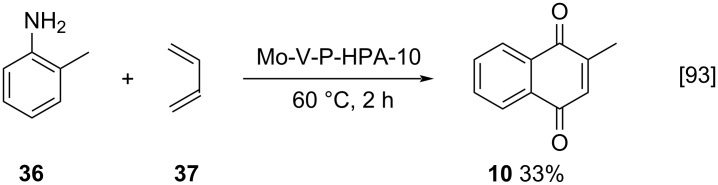
*o*-Toluidine as a dienophile in a Diels–Alder reaction for the synthesis of menadione (**10**).

In the same year, Gogin and and co-workers developed a method for the synthesis of menadione (**10**) using dienophiles from *o*-cresol or *o*-toluidine [93]. Mo-V-P-HPA-X catalysts were tested, where X is the amount of V atoms present in the molecule. All reactions led to the product in good yields. The route from *o*-toluidine (**36**) to form **10**, using Mo-VP-HPA-10 as catalyst, in the presence of 1,3-butadiene, presented the best yield (about 33%) (Scheme 9). This presented itself as a good synthetic route, considering that it used easily accessible reagents and there was no formation of polluting products.

#### Electrochemical synthesis

**Scheme 10:**
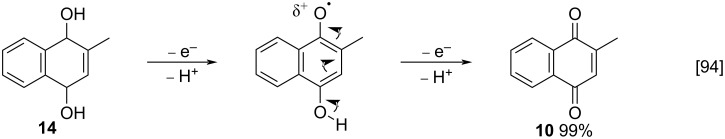
Representation of electrochemical synthesis of menadione.

Although not common, menadione (**10**) can be readily produced through electrochemical synthesis. This methodology allows the reuse of the electrolyte and demonstrates a significant substrate conversion. Raju and co-workers [94], for instance, reported the electrochemical synthesis of **10** from menadiol (**14**) using galvanostatic biphasic electrolysis (Scheme 10). For this approach, two smooth platinum foil electrodes placed 1 cm apart in the upper aqueous phase were electrolyzed galvanostatically (30 mA/cm^2^). Additionally, a NaBr solution acidified with H_2_SO_4_ was used as electrolyte. The voltage during electrolysis was 2.1 V and when the reaction was completed (charge passed of 2 F/mol), menadione (**10**) was obtained in 99% yield.

### Menadione as synthetic precursor

**Figure 4:**
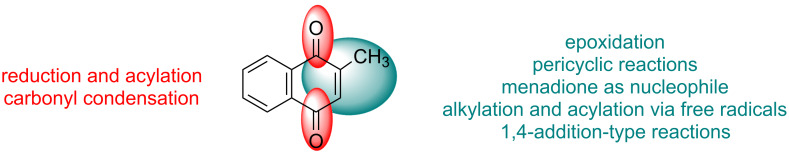
Reaction sites and reaction types of menadione as substrate.

There are many strategies available for structural modifications of menadione (**10**) and in the following section we will discuss their evolution and present those methods generally used to access menadione derivatives, to highlight the importance of this substrate for organic synthesis. The most common reaction types and reaction sites of menadione derivatization are depicted in Figure 4. The vast majority of the methods involve the unsaturated α,β-system of the naphthoquinone nucleus, due to its greater reactivity when compared to the adjacent aromatic ring, which depends on previous modifications in the menadione intermediates [95].

#### Epoxidation reactions

**Scheme 11:**
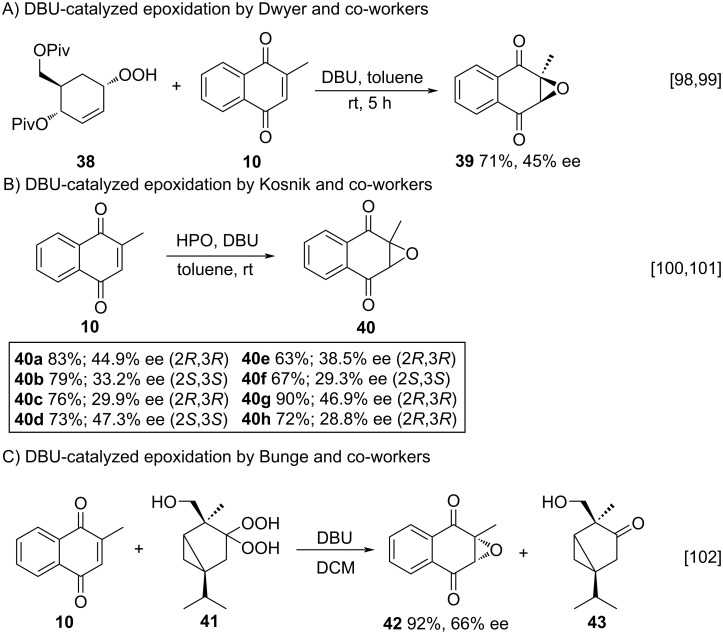
DBU-catalyzed epoxidation of menadione (**10**).

The use of menadione in the preparation of epoxides is widely reported in the scientific literature. In nature, menadione epoxides are formed through oxidation reactions in vivo, that occur in protein processes dependent on vitamin K [96–97]. Dwyer and co-workers described a procedure using sugar-derived hydroperoxides for the synthesis of epoxides in the presence of 1,8-diazabicyclo[5.4.0]undec-7-ene (DBU) as base [98–99]. The authors studied a broad range of quinone derivatives among which compound **39** was obtained through reaction of menadione (**10**) and hydroperoxide **38** in 71% yield and 45% ee (Scheme 11A). Analogously, Kosnik and co-workers described a similar epoxidation methodology using a series of pyranose-derived anomeric hydroperoxides (HPO) to obtain epoxides **40** with moderate ees (Scheme 11B) [100–101]. Bunge and co-workers used the enantiomerically pure dihydroperoxide **41** in the DBU-mediated epoxidation of menadione (**10**) for the enantioselective synthesis of epoxide **42** (92% yield and 45–66% ee) (Scheme 11C) [102].

**Scheme 12:**
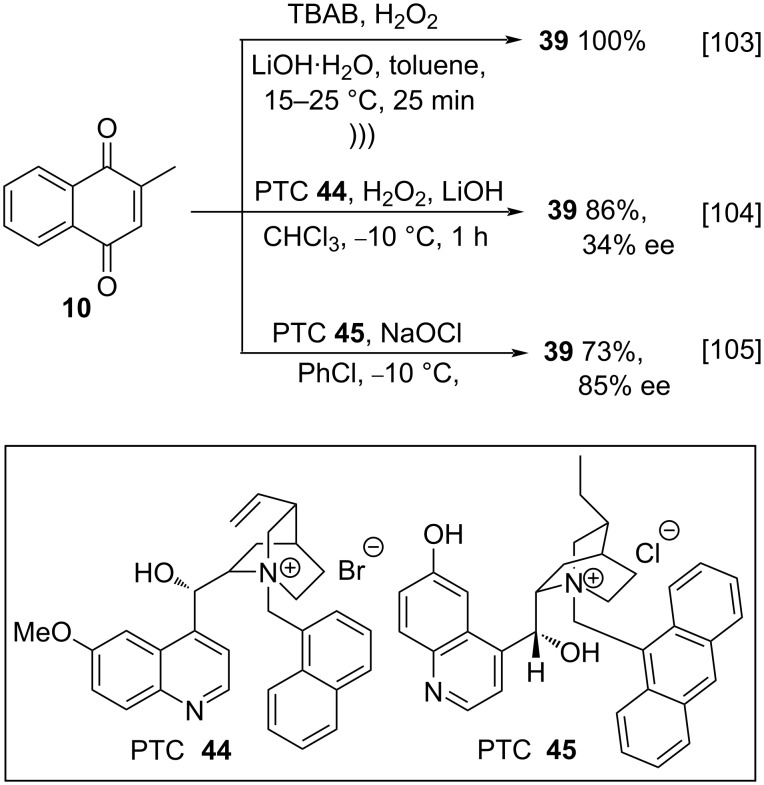
Phase-transfer catalysis for the epoxidation of menadione.

Another interesting approach to menadione epoxidation is the use of a phase-transfer catalyst (PTC). Ooi and co-workers studied the epoxidation of menadione (**10**) and other carbonylated substrates using tetrabutylammonium bromide (TBAB) as catalyst and the optimized reaction conditions involved ultrasound irradiation, obtaining quantitative yields when compared to the mechanical stirring procedure, thus demonstrating the best efficiency of the method [103]. In 2002, Arai and co-workers reported studies involving epoxidation reactions of menadione (**10**) using H_2_O_2_ as oxidant and a chiral salt derived from cinchonine PTC **44** as catalyst. Despite of good yields, the method did not demonstrate good enantioselectivity results [104]. Berkessel and co-workers, in turn, described the use of asymmetric Weitz–Scheffer-type epoxidation of menadione (**10**), mediated by cinchona alkaloid PTC **45**, showing high enantioselectivity (85% ee) (Scheme 12) [105].

**Scheme 13:**
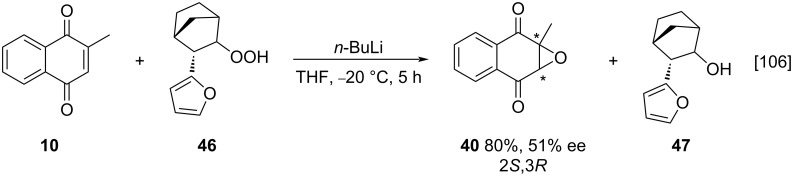
Menadione epoxidation using a hydroperoxide derived from (+)-norcamphor.

Exploring a different epoxidation reaction approach, Lattanzi and co-workers reported a methodology using a (+)-norcamphor hydroperoxide **46**, to generate the menadione-derived epoxide **40** in 51% ee, under optimized reaction conditions employing *n*-BuLi/THF [106]. The method proved to be effective at recovering approximately 95% of the enantiopure alcohol **47**. This allowed the alcohol’s effective reconversion to hydroperoxide **46** and proved to be a useful method for the enantioselective epoxidation of menadione (**10**) (Scheme 13).

#### Pericyclic reactions

The Diels–Alder reaction, among pericyclic reactions, is a very important synthetic approach to obtain several molecular scaffolds, including naturally occurring molecules, drugs, polymers, and heterocycles with promising biological activity. Especially, Diels–Alder reactions involving quinones and dienes as starting materials allow for the synthesis of more complex molecules such as natural products. Within this scope, the menadione (**10**) molecule has been explored as substrate for this versatile reaction.

**Scheme 14:**
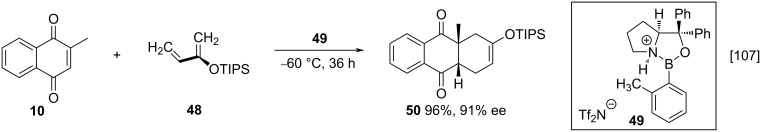
Enantioselective Diels–Alder reaction for the synthesis of asymmetric quinone **50** catalyzed by a chiral oxazaborolidinium cation.

Ryu and co-workers described an enantioselective and structurally selective Diels–Alder reaction for the synthesis of asymmetric compound **50** catalyzed by a chiral oxazaborolidinium cation (**49**) [107]. This type of catalyst has been used in several Diels–Alder reactions proving to be an excellent choice for highly enantioselective reactions [108]. For instance, the reaction of menadione (**10**) with 2-triisopropylsilyloxy-1,3-butadiene (**48**) gave compound **50** in 96% yield and 91% ee (Scheme 14).

In 2006, Nishimoto and co-workers described an interesting application of a Diels–Alder reaction conducted, among others, in water and fluorous solvents [109]. Especially, the employment of water as solvent has gained great attention in the last few years in organic synthesis due to its physical and chemical properties. The authors studied the Diels–Alder reaction between menadione (**10**) and 2,3-dimethyl-1,3-butadiene (**51**) and it was possible to draw a comparison between the reported properties for this type of solvent (Table 4). The studies showed that the emulsion, prepared by sonication, of an equimolar mixture of lithium perfluorooctane sulfonate (LiFOS) and perfluorohexane (PFH) in aqueous medium resulted in a significant increase of the reaction rate, when compared to other reaction conditions [109]. As an example, using an equimolar ratio of 500 mM, the Diels–Alder reaction achieved 98% and 2% recovery of starting material after 72 h, at an estimated product generation rate of 142.5 μM/h. In this context, it was possible to apply a green methodology without the application of heating or the use of Lewis acids, contributing significantly to the progress of the synthesis studies in aqueous medium.

**Table 4:** Media effect in the initial rates of Diels–Alder reaction between menadione and 2,3-dimethyl-1,3-butadiene.

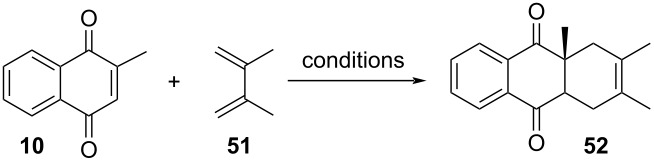			

Conditions (organic solvents)	Reaction rate (µM/h)	Conditions (aqueous)	Reaction rate (µM/h)

perfluorohexane (PFH)	0.25	aq SDS (100 mM)	3.87
*n*-hexane	0.38	aq TFE (100 mM)	3.80
diethyl ether	1.00	aq LiOTf (100 mM)	1.15
methanol	1.27	aq LiOS (100 mM)	2.35
acetonitrile	1.60	aq LiFOS (100 mM)	11.6
dichloromethane	3.16	neat	<0.2
toluene	0.73	LiFOS/PFH in water (500 mM/500 mM)	142.5
water	1.40		

**Scheme 15:**
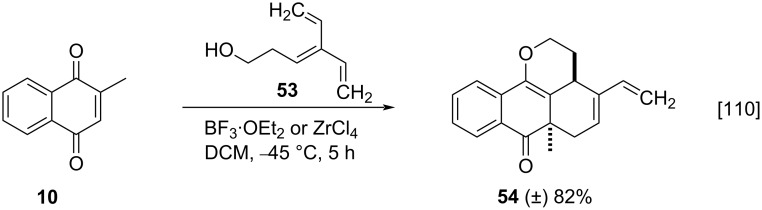
Optimized reaction conditions for the synthesis of anthra[9,1-*bc*]pyranone.

The Diels–Alder reaction using menadione (**10**) was also studied by Bendiabdellah and co-workers, who reported the intramolecular Diels–Alder domino reactions promoted by Lewis acids [110]. The reaction involving menadione (**10**) and excess of triene **53** was carried out using boron trifluoride diethyl etherate or zirconium(IV) tetrachloride to furnish product **54** in good yield (Scheme 15).

**Scheme 16:**
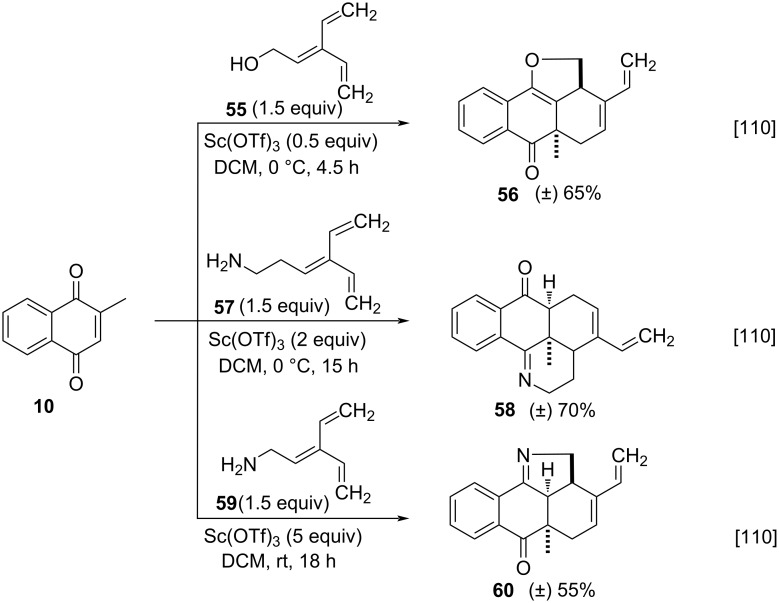
Synthesis of anthra[9,1-*bc*]furanone, anthra[9,1-*bc*]pyridine, and anthra[9,1-*bc*]pyrrole derivatives.

The same group also explored the Diels–Alder reaction of menadione (**10**) with trienes **55**, **57**, and **59** (Scheme 16) [110]. The scandium(III) triflate-catalyzed reaction showed the best results in terms of performance, producing furanone **56** in 65% yield. Additionally, this study was also extended to the synthesis of hetero-tetracyclic derivatives containing endocyclic nitrogen atoms. The best result was obtained using triene **57**, and 2.0 equivalents of scandium(III) triflate, affording compound **58** in 70% yield. Finally, the optimized conditions were employed to react menadione (**10**) with the triene **59**, using 5.0 equivalents of scandium(III) triflate, forming compound **60** in 55% yield.

**Scheme 17:**
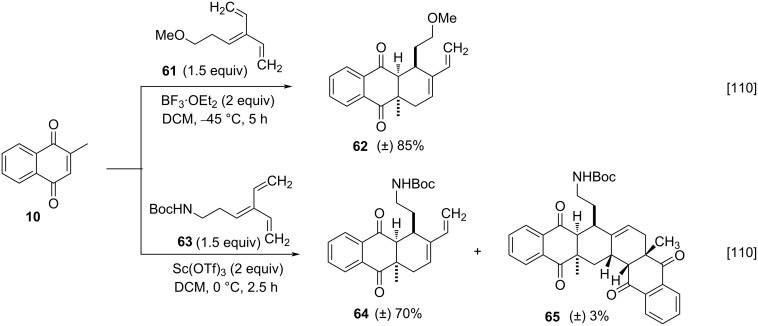
Synthesis of derivatives employing protected trienes.

Additional studies were made, using protected trienes, and in Scheme 17 below the optimized reaction conditions are shown for the synthesis of compounds **62** and **64** (Scheme 17) [110].

**Scheme 18:**
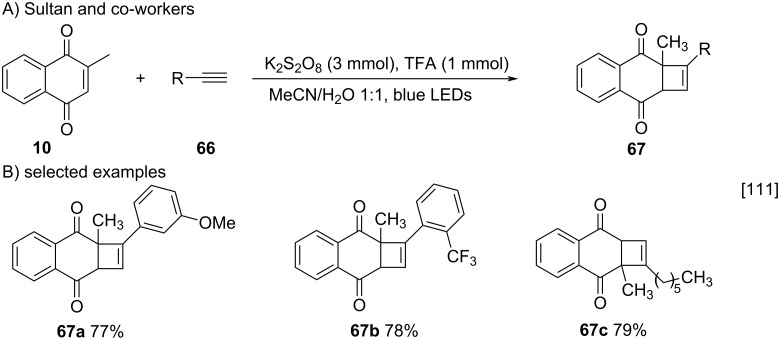
Synthesis of cyclobutene derivatives of menadione.

In 2019, Sultan and co-workers described a methodology for the synthesis of quinone derivatives using a combination of potassium persulfate, trifluoroacetic acid (TFA), and blue-LED light [111]. Under these conditions, menadione (**10**) and terminal alkynes **66** underwent a [2 + 2] cycloaddition reaction generating compounds containing cyclobutene rings (**67a–c**), that are important precursors in natural products syntheses. It is important to note that the choice of the blue-LED source was made after preliminary studies demonstrated the occurrence of reactions with benzoquinones using the compact fluorescent lamp (Scheme 18).

#### Reduction and acylation reactions

Menadione reduction reactions are one of the most important types of reactions and are directly related to some of its characteristic properties, such as the biological redox cycle. The main and most common menadione reduction product is menadiol or vitamin K_4_, followed by its dialkyl ether and diacyl derivatives.

**Scheme 19:**
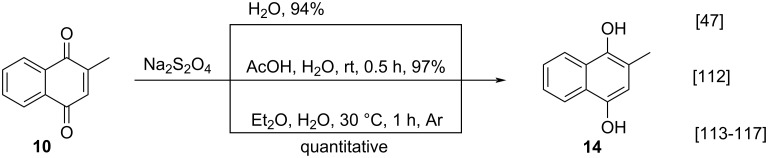
Menadione reduction reactions using sodium hydrosulfite.

Menadione can be easily converted to menadiol (**14**) by reduction with sodium dithionite, as first described by Fieser (Scheme 19) [47]. The reaction was carried out in a separatory funnel to which was added menadione (**10**), sodium hydrosulfite, and water [47]. The mixture was shaken for a few minutes until the solution passed through a brown phase and became yellow. Despite of being an old method, it is very efficient and widely used with some adaptations. In 2003, Ito and co-workers also used sodium dithionite to obtain menadiol (**14**) from **10**. However, in this case, the authors mixed menadione (**10**) and sodium hydrosulfite in acetic acid and water to obtain **14** in 97% yield [112]. More recently, still using sodium dithionite, Suhara and co-workers reported in their various works on the synthesis of vitamin K analogues, the use of menadione (**10**) to obtain **14** [113–117]. In these works, menadione (**10**) was reduced by using an aqueous 10% sodium dithionite solution in diethyl ether to furnish alcohol **14** in a quantitative yield.

**Scheme 20:**
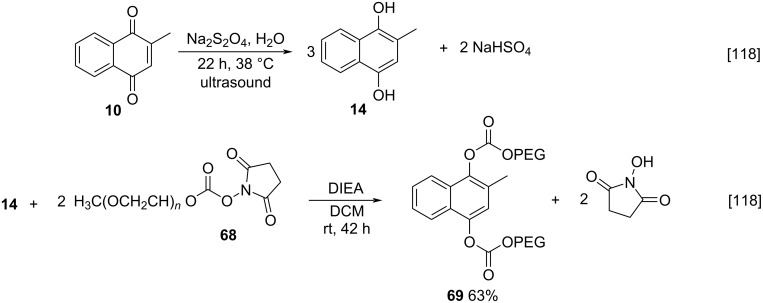
Green methodology for menadiol synthesis and pegylation.

Still about reduction reactions, Niemczyk and Van Arnum described a green methodology for reduction of menadione (**10**), during the pegylation of **14** to improving the solubility of the studied compounds [118]. The authors reduced **10** with sodium dithionite under ultrasound irradiation, generating the reduced adduct in 79% yield (Scheme 20). Depending on the type of solvent used, the yield may vary due to oxidation of the alcohol **14** back to **10** because of its low stability in solution. The pegylation strategy involved monomethoxypoly(ethyleneglycol)succinimide carbonate (mPEG-SC, **68**), giving the pegylated product and *N*-hydroxysuccinimide (NHS) as the sole byproduct. The latter can be recycled again to the pegylation reagent. This study showed better results when compared to the methodology for a phosphorylation of **14** developed by Fieser [119], a procedure carried out in two steps whose main difficulties are the separation of pyridine byproducts and inorganic phosphate (Scheme 20).

**Scheme 21:**
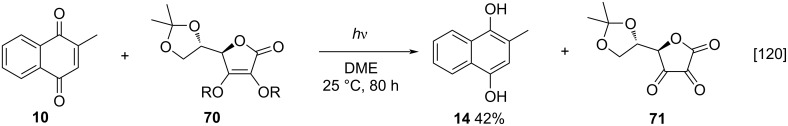
Menadione reduction by 5,6-*O*-isopropylidene-ʟ-ascorbic acid under UV light irradiation.

Kulkarni and co-workers reported a method for menadione reduction mediated by 5,6-*O*-isopropylidene-ʟ-ascorbic acid (**70**, R = H) under UV light irradiation [120]. Initial studies were carried out using lawsone (**4**) and after optimization of the reaction conditions, it was extended to other quinones, including menadione (**10**). The best conditions were 1,2-dimethoxyethane (DME) as solvent, temperature 25 °C, under ultraviolet light irradiation (125 W lamp) using a Pyrex filter in an immersion-well photoreactor. It was observed that the presence of free hydroxy groups in **70** was essential for the quinone reduction reaction to occur, when compared to the 2,3-di-*O*-methylated derivative (**70**, R = Me). Under the reported conditions, the reduction of menadione (**10**) gave compound **14** in 42% yield after 80 hours (Scheme 21).

**Scheme 22:**
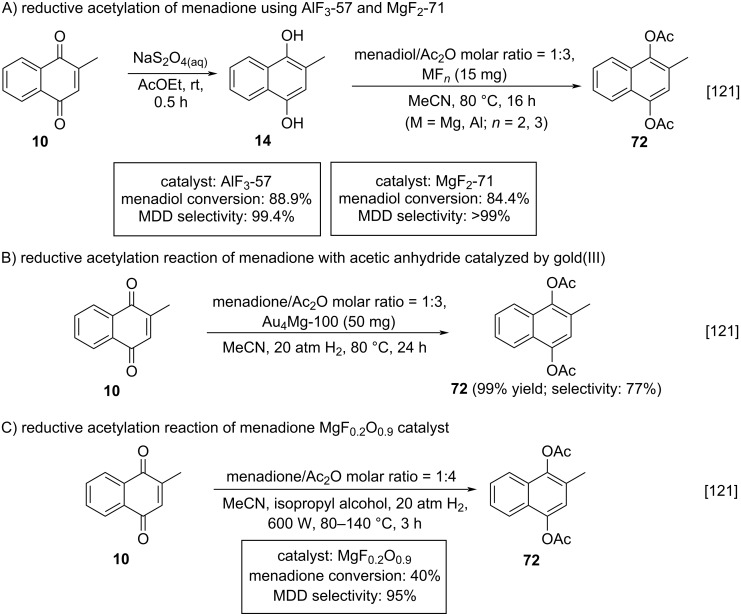
Selected approaches of menadione hydroacetylation to diacetylated menadiol.

Dobrinescu and co-workers studied, besides acetylation reactions, the hydroacetylation of menadione for the synthesis of diacetylated menadiol derivatives through heterogeneous catalysis [121]. The first methodology involved the reduction of menadione (**10**) to menadiol (**14**), with sodium dithionite, followed by hydroacetylation of **14** with acetic anhydride, using nanoscopic acidic hydroxylated metal fluorides MF*_n_*_-_*_x_*(OH)*_x_* (M = Mg, Al; *n* = 2, 3; *x* < 0.1) as catalysts. This type of catalysts has a huge acidic versatility, once they can behave as Brønsted or Lewis acids. The reductive acetylation of **10** occurred in two steps and at high selectivity conversion rates when using AlF_3_-57 and MgF_2_-71 (Scheme 22A). The second proposal explored the reductive acetylation reaction of menadione (**10**) with acetic anhydride catalyzed by gold(III) deposited on the qualified metallic fluorides. The deposition of gold on metallic fluorides allowed the one-pot hydroacetylation of menadione (**10**) to diacetylated menadiol **72**, while the deposition of gold on silica allowed only the hydrogenation of **10** to **14** (Scheme 22B). The authors also observed that catalysis by hydroxylated fluorides led to a higher reaction speed when compared to the use of gold-impregnated catalysts, indicating that gold(III) impregnation blocks are part of the active sites for menadiol acetylation. The third approach was based on the Meerwein–Ponndorf–Verley (MPV) reduction coupled with an acetylation reaction, in combination with acid–base magnesium oxide fluoride (MgF*_x_*O*_y_*) as catalyst. The MPV reduction is known as being highly selective for the reduction of C=O bonds [122]. The best methodology for this third catalytic approach was based on the MgF_0.2_O_0.9_ catalyst, with the reaction time being optimized from 48 h to 3 h, which resulted in an increase of menadione (**10**) conversion (40%) and also in the reaction selectivity (95%) (Scheme 22C).

**Scheme 23:**
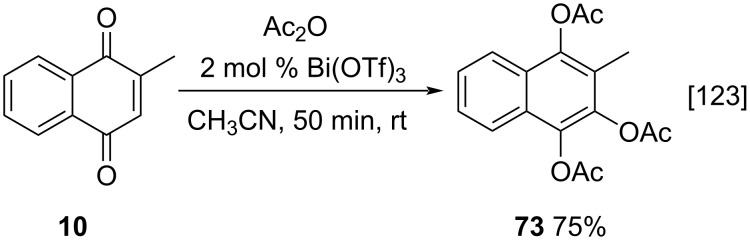
Thiele–Winter reaction catalyzed by Bi(OTf)_3_.

Another approach involving menadione acetylation was reported by Yadav and co-workers, who described a methodology for the synthesis of acetylated quinone derivatives catalyzed by bismuth(III) triflate [123], through an adapted Thiele–Winter acetoxylation reaction. The standard procedure involved the use of acetic anhydride and sulfuric acid catalysis. However, the use of sulfuric acid, a strong acid and oxidizing agent, can produce tar in some cases. In order to get around this problem a strategy applying Lewis acids can be used. Among the options, bismuth triflate is a catalyst, with the additional advantages of low-cost and easy preparation from commercially available bismuth oxide and triflic acid. Quinone acylation reactions took place under mild conditions using acetic anhydride and 2 mol % bismuth(III) triflate catalyst and compound **73** was obtained in 75% yield (Scheme 23).

#### Carbonyl condensation

**Scheme 24:**
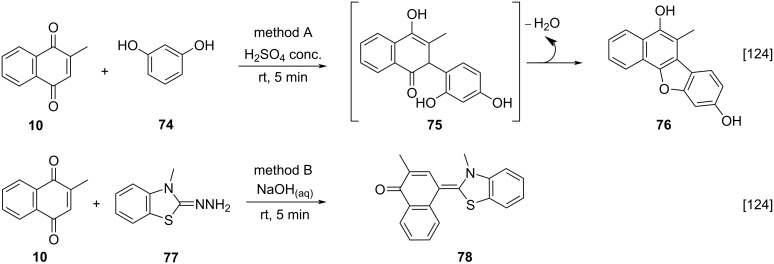
Carbonyl condensation of menadione using resorcinol and a hydrazone derivative.

Another possible derivatization involves transformations at the carbonyl groups in menadione that can be achieved through the addition of nucleophiles to the carbonyl group at position C-4. In this context, Nagaraja and co-workers reported the condensation of menadione (**10**) under different mild conditions, during the development of analytical methods for determining **10** in pharmaceutical preparations [124]. In method A, menadione (**10**) was treated with concentrated sulfuric acid and resorcinol (**74**), generating the intermediate **75** which underwent intramolecular condensation, to furnish **76**. In method B, menadione (**10**) was treated with 3-methyl-2-benzothiazolinone hydrazone (**77**) in alkaline medium forming diazocompounds as addition products, which spontaneously lose N_2_ to form product **78** (Scheme 24).

**Scheme 25:**
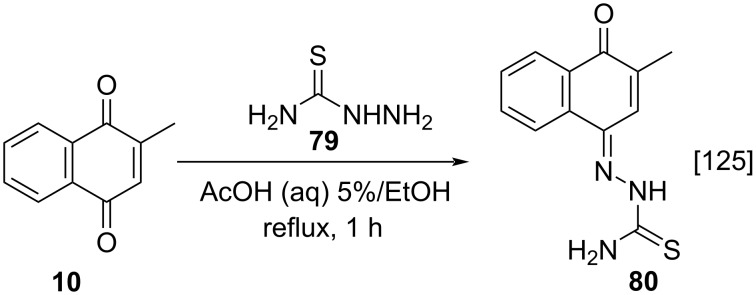
Condensation reaction of menadione with thiosemicarbazide.

Tang and co-workers described the synthesis of thiosemicarbazone **80** from menadione (**10**) through a condensation reaction with thiosemicarbazide (**79**), which was used as a ligand in the synthesis of metal complexes using different transition metals, in refluxing ethanol (Scheme 25) [125].

**Scheme 26:**
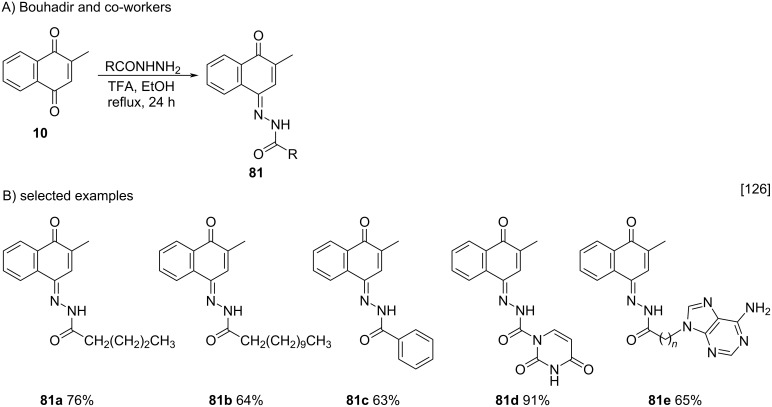
Condensation reaction of menadione with acylhydrazides.

Examining a broad range of hydrazides in the condensation reaction with menadione, Bouhadir and co-workers reported the synthesis of various menadione acylhydrazone derivatives [126]. In this work, various acylhydrazides prepared by reaction of hydrazine hydrate with different esters were reacted with menadione (**10**) in trifluoroacetic acid under ethanol reflux conditions (Scheme 26) to synthesize acylhydrazones **81** in 63–91% yield. In view of the different structures that compounds **81a**–**e** could adopt, after analysis by 2D NMR-NOESY spectra, it was found that all products were obtained as *E*-geometrical isomers and *trans*-conformers.

#### Menadione as a nucleophile

**Scheme 27:**
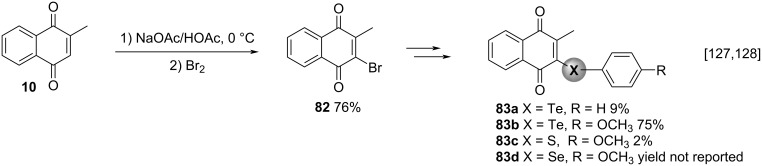
Menadione derivatives functionalized with organochalcogens.

Electron-rich 1,4-naphthoquinones, such as 2-hydroxy-, 2-amino-, and 2-alkylnaphtho-1,4-quinones may react as nucleophiles. Hence, menadione (**10**) can act as a nucleophile in, for example, bromination reactions [127] and aldol-type reactions with aldehydes and ketones [128]. In this context, Fry and co-workers explored the electrophilic substitution reaction to synthesize 2-methyl-3-bromonaphthalene-1,4-dione (**82**), an important intermediate used for the synthesis of naphthoquinones functionalized with organochalcogens [127]. Compound **82** was obtained by treating menadione (**10**) with molecular bromine in the presence of sodium acetate and acetic acid in 76% yield. With brominated compound **82** at hands, the authors obtained four menadione derivatives **83a**–**d** functionalized with organochalcogenic moieties after treatment of compound **82** with the respective ditellurides, a disulfide and a diselenide (Scheme 27).

**Scheme 28:**
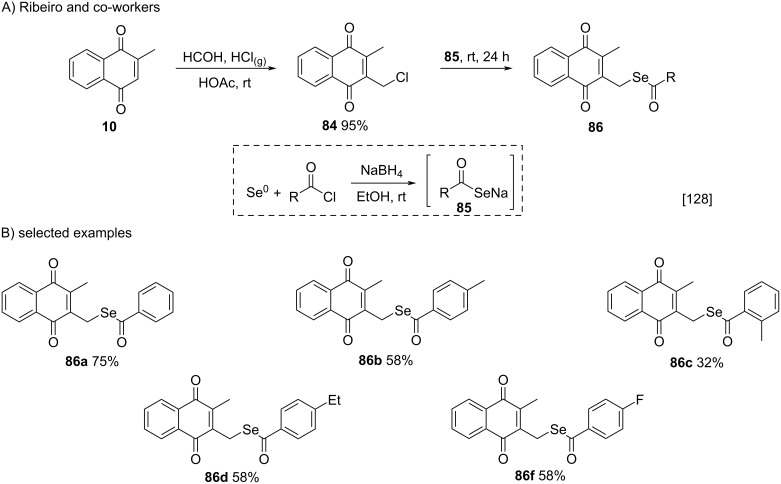
Synthesis of selenium-menadione conjugates derived from chloromethylated menadione **84**.

Recently, Ribeiro and co-workers used menadione as a nucleophile for the synthesis of 3-chloromethylated menadione **84**, a key intermediate used to prepare selenium-menadione conjugates **86** [128]. In this work, compound **84** was prepared through the reaction of menadione (**10**) with formaldehyde in the presence of gaseous HCl bubbled into the reaction medium [129–130]. Then, the chloromethyl derivative **84** was treated with diselenides, generated in situ from the reaction between Se^0^, NaBH_4_ and different acid chlorides, to form conjugates **86** in 24–75% yields (Scheme 28).

#### Alkylation and acylation by free radicals

**Scheme 29:**
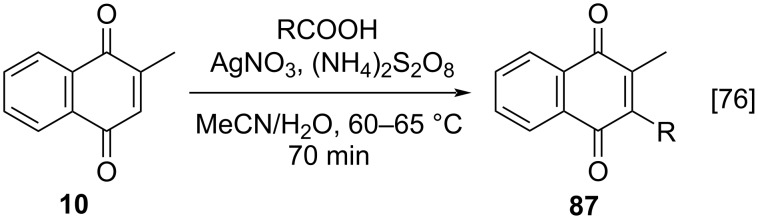
Menadione alkylation by the Kochi–Anderson method.

One of the largest groups of reactions that use menadione (**10**) as substrate comprises free radical alkylation and acylation reactions. The most useful alkylation approach is the Kochi–Anderson method [76] or (Jacobsen–Torssell method [77]) where **10** reacts with a carboxylic acid in the presence of silver(I) nitrate and ammonium or potassium peroxydisulfate (Scheme 29).

**Scheme 30:**
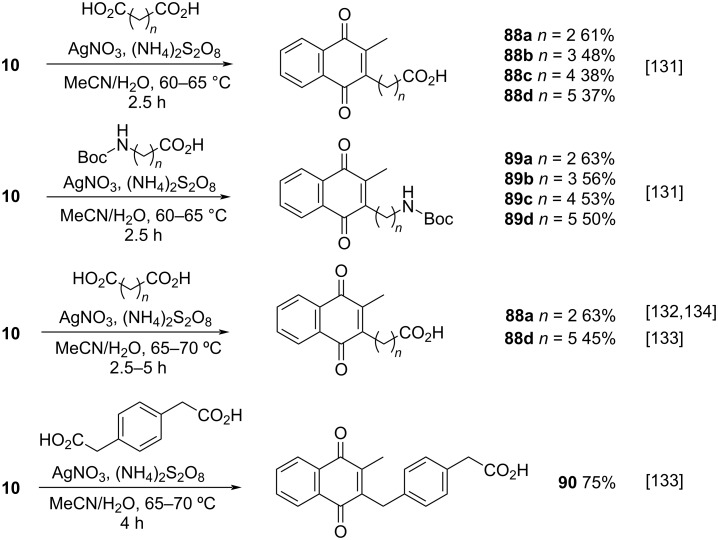
Menadione alkylation by diacids.

In the past twenty years, several menadione alkylation studies have been carried out based on the Kochi–Anderson method [131–141]. In 2001, Salmon-Chemin and co-workers described the preparation of alkylated compounds **89** and **90** via oxidative decarboxylation of diacids and *N*-Boc-protected amino acids β-alanine, γ-aminobutyric acid, 5-aminovaleric acid, and 6-aminocaproic acid [131]. The compounds were obtained in moderate yields (37–63%), and a decreased yield was observed with an increase of the aliphatic chain length, which was more accentuated for the diacid derivatives, and more subtle for the amino acid derivatives (Scheme 30). The same methodology was applied during the synthesis of oligopeptides linked to **10** (Scheme 30) [132–134].

In detailed studies, initiated by Commandeur and co-workers [135] and expanded by Naturale and co-workers [139], the alkylation capabilities of menadione (**10**) were evaluated, exclusively, with several amino acid types by Kochi–Anderson radical decarboxylation [76]. In the studies developed by Naturale’s group, α-, β-, and γ-amino acids of linear and branched chains were used, as well as different amine protection groups (Table 5). The results revealed that the functionalization of naphthoquinones by a radical addition of decarboxylated α-, β- and γ-*N*-protected amino acids was possible. However, the high conversion rates of the reagents to the desired products were not reflected in the isolated product yields, which was attributed to the workup and purification processes. It was also possible to demonstrate a moderate influence of the *N*-protecting group on the reaction outcome, although electronic effects can be considered to play a role, especially with substituted α-amino acids.

**Table 5:** Menadione alkylation with amino acids by Naturale’s group.

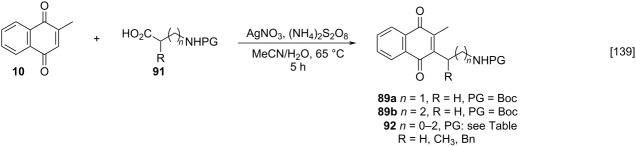					

Entry	Amino acid	Product	Protective group	Conversion (%)	Yield (%)

1	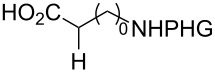	**92a**	Boc	74	40
		**92b**	Troc	89	58
		**92c**	Ac	98	49
		**92d**	TFA	100	42
2	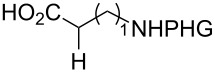	**89a**	Boc	96	58
		**92e**	Troc	100	42
		**92f**	Ac	57	13
		**92g**	TFA	100	51
3	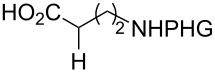	**89b**	Boc	88	61
		**92h**	Troc	86	60
		**92i**	Ac	75	54
		**92j**	TFA	98	53
4	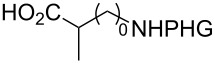	**92k**	Boc	0	0
		**92l**	Troc	35	18
		**92m**	Ac	29	16
		**92n**	TFA	92	27
5	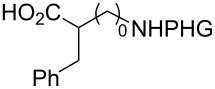	**92o**	Boc	0	0
		**92p**	Troc	42	34
		**92q**	Ac	24	24
		**92r**	TFA	29	29
6	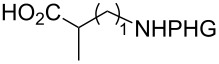	**92s**	Boc	100	50
		**92t**	Troc	100	55
		**92u**	Ac	93	57
		**92v**	TFA	100	39

**Scheme 31:**
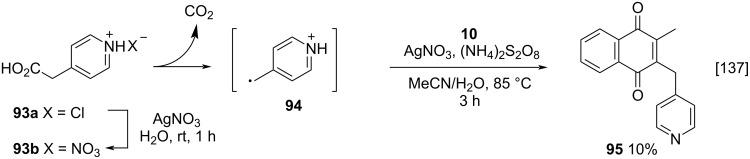
Menadione alkylation by heterocycles-substituted carboxylic acids.

Lanfranchi and co-workers also studied the menadione (**10**) alkylation by oxidative decarboxylation using carboxylic acids containing nitrogenous heterocycles as substituents, and achieved very interesting results [137]. The authors observed that the desired product **95** was obtained with very low yield, due to the competition between the Kochi–Anderson [76] reaction and the Minisc reaction (Scheme 31) generating a mixture of polymeric pyridine derivatives [142]. The Minisci reaction [142] is a nucleophilic radical substitution to an electron-deficient aromatic compound, in the presence of silver(I) nitrate, ammonium persulfate, and heat, reaction conditions that are very similar to those of the Kochi–Anderson procedure. Under these conditions, the γ-picoline radical preferentially reacts with itself (or the starting pyridylacetic acid) rather than with menadione (**10**) [137].

**Scheme 32:**
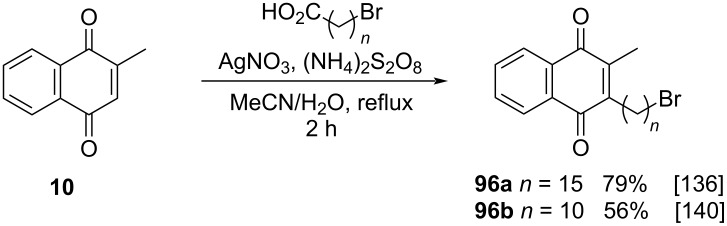
Menadione alkylation by bromoalkyl-substituted carboxylic acids.

The Kochi–Anderson reaction can also be used for the alkylation of menadione with bromoalkyl-substituted carboxylic acids as described by Terasaki and co-workers [136] and Liu and co-workers [140]. The alkylation products **96a**,**b** were obtained in good yields, demonstrating a greater resistance of this type of compounds to the workup and purification processes, when compared to derivatives with other types of long chain acids and substituents in the methyl terminal (Scheme 32).

**Scheme 33:**
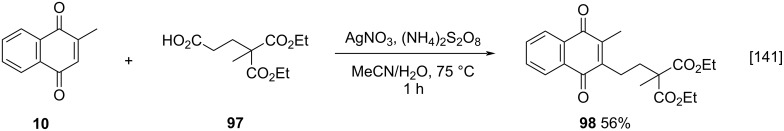
Menadione alkylation by complex carboxylic acids.

An example for the alkylation of menadione by the Kochi–Anderson method with a complex carboxylic acid was described by Goebel and Barany in the synthesis of **98**, a human metabolite formed from vitamin K with biological activity [141]. In this work, a carboxylic acid derived from diethyl methylmalonate was used, and the product **98** was obtained in 56% yield, which is comparable to yields found with short chain diacids, as previously mentioned (Scheme 33).

Some variations of the Kochi–Anderson method have also been described in the literature, such as the methods reported by Gutiérrez–Bonet and co-workers [143] and Sutherland and co-workers [144], both with no use of silver as radical generator, the method reported by Liu and co-workers [145], using cyclic amines as alkylating agents, and the method described by Pandaram [146], using silver salt and TBHP as oxidizing agent.

**Scheme 34:**
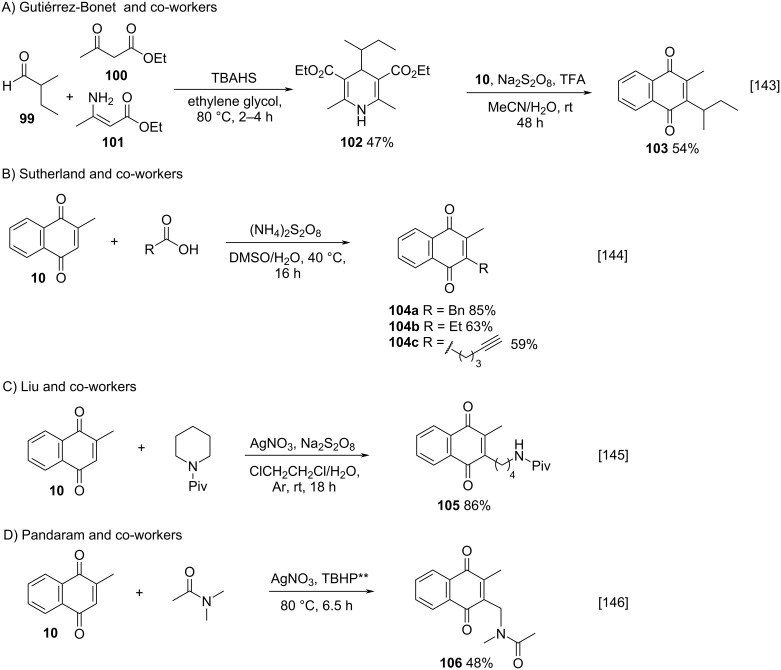
Kochi–Anderson method variations for the menadione alkylation via oxidative decarboxylation of carbonyl substrates.

In the reaction described by Gutiérrez-Bonet [143], the authors used persulfate as oxidizing agent, trifluoroacetic acid as menadione-activator and 1,4-dihydropyridine **102**, which was readily prepared from aldehyde **99** in one step, to achieve homolysis [143]. The advantage of the method is the absence of a noble metal salt and milder reaction conditions; however, it presents also some disadvantages such as a longer reaction time and the need of pre-functionalized aldehydes. In turn, Sutherland and co-workers [144] described the menadione (**10**) alkylation with persulfate, alkylcarboxylic acids, and dimethyl sulfoxide (DMSO) at 40 °C, in a process without metals, photocatalysts, light or pre-functionalized alkyl substrates (Scheme 34B). This study demonstrated that the silver salt is not essential for the alkylation to occur. Compounds **104a**–**c** were obtained in good yields, similar to the yields obtained by the original process (Scheme 34B). The success of this procedure is related to the easier decomposition of persulfate to form sulfate radicals (SO_4_^•−^) in DMSO. In their work, Liu and co-workers described the access to distal aminoalkyl-substituted menadione **105** by silver-catalyzed site-selective ring-opening and C–C-bond functionalization of the cyclic amine in good yields (Scheme 34C) [145]. This approach overcame other methods’ problems, such as multi-stage transformations or the use of short-chain amino acids. Recently, Pandaram and co-workers demonstrated that menadione (**10**) also could be amidoalkylated using silver(I) nitrate – *tert*-butyl hydroperoxide in *N,N*-dimethylacetamide as alkylating agent and solvent (Scheme 34D) [146]. This was the first reported synthesis of several amidoalkylated quinones that were obtained in moderate and good yields. No pre-functionalization of starting materials was required, and only nonhazardous reagents were used.

Despite its widespread use, the Kochi–Anderson method for the alkylation of menadione has as its main limitation the exclusive use of carboxylic acids as alkyl chain source, thus restricting the substrate scope. In this context, several approaches have been developed to replace carboxylic acids with a different radical source, such as alkyl halides, alkanes or activated alkenes, in conjunction with transition-metal catalysis or metal-free processes.

**Scheme 35:**
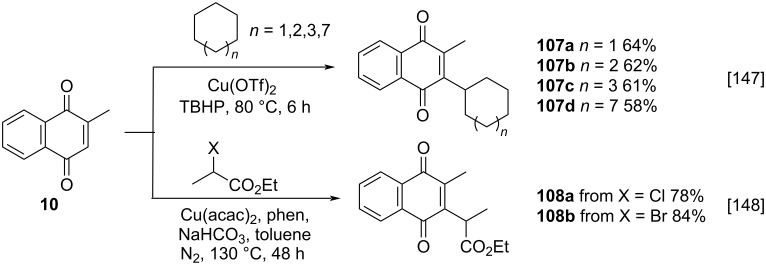
Copper-catalyzed menadione alkylation via free radicals.

In a work published by Baral and co-workers, the unprecedented Csp^2^–Csp^3^ alkylation of menadione (**10**) with medium and large-size cyclic alkanes was achieved by the combination of copper(II) triflate and *tert*-butyl hydroperoxide (Scheme 35) [147]. The products **107a**–**d** were obtained in 58–64% yield range. This is a one-step process with no need of activated alkylating substrates. In turn, Li and Yang also reported the use of copper as alkylation promoter without the use of an oxidizing agent. However, in their method functionalized alkyl halides and high temperatures were used, to obtain compound **108b** in 84% yield (Scheme 35) [148].

**Scheme 36:**
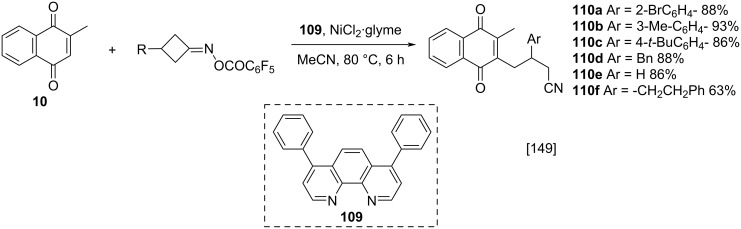
Nickel-catalyzed menadione cyanoalkylation.

Gu and co-workers explored the reactivity of cyclobutanone oximes as alkylation substrates for menadione (**10**), in a reaction catalyzed by nickel and oxidizing agents free [149]. With this method, it was possible to obtain cyanoalkylated compounds **110** in excellent yields, from a wide range of cyclobutanone oximes with aryl, benzyl or alkyl groups (Scheme 36).

**Scheme 37:**
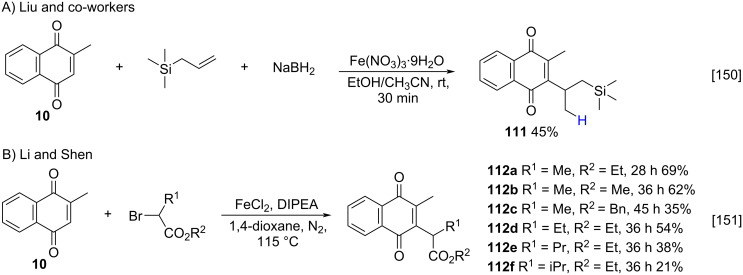
Iron-catalyzed alkylation of menadione.

Recently, the use of iron as catalyst in the alkylation reaction of menadione via free radicals has also been reported [150–151]. Iron has many advantages, such as its high abundance, low-cost, and low toxicity to humans and the environment, that make iron quite attractive to be used in synthetic processes. Liu and co-workers described a radical alkylation of menadione (**10**) with an olefin as the radical precursor, during the iron(III)-mediated C–H conversion of quinones with non-activated alkenes (Scheme 37A) [150]. In their work, Li and Shen used a general iron-catalyzed protocol for the synthesis of alkylated quinones, including menadione, with alkyl bromides as alkylating reagents, with a broad substrate scope, densely functional group tolerance, and good yields (Scheme 37B) [151]. A common advantage to both methods is the absence of oxidizing agents to generate the radical species. However, the method described by Li requires harsher conditions, such as temperatures above 100 °C and reaction times longer than 24 hours, when compared to the method developed by Liu.

Other menadione alkylation methods, by free radicals, which do not involve the Kochi–Anderson procedure [76], its adaptations, or catalysis mediated by transition metals have also been reported. These methods include alkylation by thermal decomposition of diacid peroxides [152], the use of organotellurium compounds [153–154], and perfluoroalkylation from perfluoroalkyl radicals [155].

**Scheme 38:**
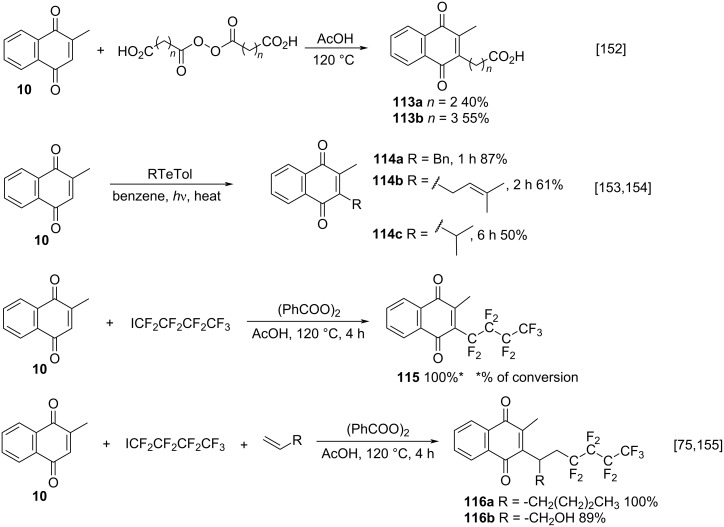
Selected approaches to menadione alkylation.

Boudalis and co-workers reported a selective alkylation method for menadione with radicals generated from the thermal decomposition of diacyl peroxides **113a**,**b** (Scheme 38) [152], as an adaptation of the route developed by Fieser [156]. Yamago and co-workers also described the alkylation of menadione, during the synthesis mediated by radicals of quinones substituted with organotellurium compounds (Scheme 38) [153–154]. A very specific type of radical alkylation of menadione was described by Antonietti [75] for the synthesis of perfluoronaphthoquinones from perfluoroalkyl radicals, with or without alkenes presence (Scheme 38). In these studies, it was observed that in the absence of alkenes, perfluoroalkylation occurs directly on the menadione C-3 carbon, generating product **115**. On the other hand, in the presence of a terminal alkene, perfluoroalkylation occurs first on the alkene and then the obtained free radical reacts with menadione, leading to products **116a**,**b**. The same results were obtained for Sansotera and co-workers who used perfluorodiacyl peroxides as alkylating agents [155].

**Scheme 39:**
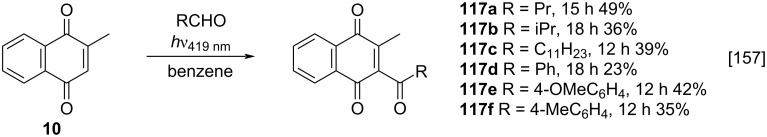
Menadione acylation by photo-Friedel–Crafts acylation reported by Waske and co-workers.

In contrast to alkylation, the radical acylation of menadione is not very common. In one of the few reports that exclusively is dedicated to the study of the radical acylation of menadione, Waske and co-workers described a versatile method for the preparation of photoacylated menadione products from aldehydes using free radical conditions [157], also called photo-Friedel–Crafts acylation, a term introduced by Oelgemöller [158–161]. According to this protocol, a mixture of menadione (**10**) and an aliphatic or aromatic aldehyde in excess, in benzene, is irradiated under direct excitation conditions (λ_max_ = 419 nm). The acylated menadione derivatives **117a**–**f** were obtained in moderate yields (Scheme 39).

**Scheme 40:**
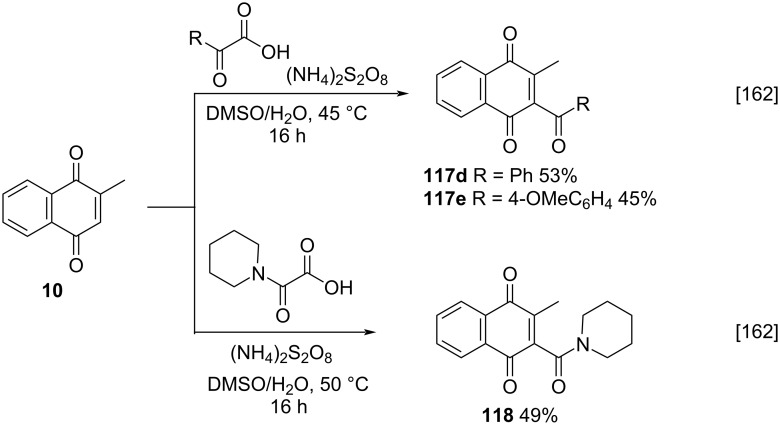
Menadione acylation by Westwood procedure.

Westwood and co-workers developed a decarboxylative acylation for the direct C–H acylation and carbamoylation of heterocycles, including menadione, under metal-, photocatalyst-, and light-free conditions [162] based on the method developed by Minisci [142]. The reaction occurs between menadione (**10**) and acyl radicals derived from α-keto acids and alkyl-substituted oxamic acid in the presence of persulfate in DMSO [142], providing acylated products **117d**,**e** and **118**, respectively, in moderate yields (Scheme 40).

**Scheme 41:**
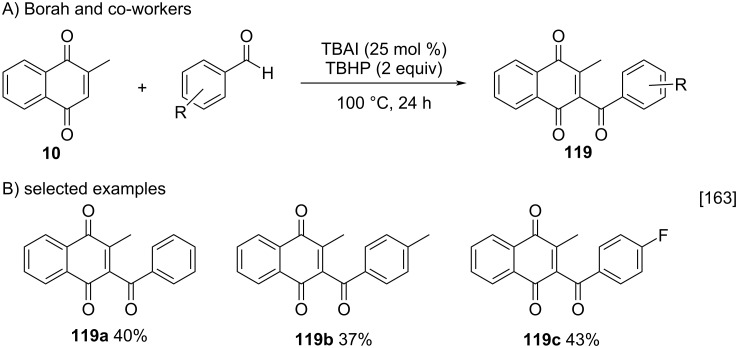
Synthesis of 3-benzoylmenadione via metal-free TBAI/TBHP system.

Borah and co-workers described a methodology for the free radical benzoylation of 2-substituted-1,4-naphthoquinones, such as menadione, as an alternative approach to the use of organometallic reagents [163]. Considering some limitations of the methods commonly used in acylation reactions via free radicals, such as the use of metallic catalysts, long reaction times, and acyl/benzoyl source, in Borah’s work the acylation of menadione via benzoyl radicals was performed using the metal-free tetra-*n*-butylammonium iodide/*tert*-butyl hydroperoxide (TBAI/TBHP) system [163]. Under optimized conditions the three benzoylated compounds **119a–c** were obtained with 37–43% yield (Scheme 41). The modest yields of the menadione derivatives, when compared to halogenated derivatives, can be explained by the interaction of the methyl group with the TBAI/TBHP system [163].

#### 1,4-Addition reactions

Menadione behaves like a typical Michael acceptor in the presence of nucleophiles, such as amines and thiols, and the addition of the nucleophile occurs at the C-3 carbon, which is less steric impeded and more electrophilic [164]. The formed adduct is a naphthohydroquinone which is then oxidized, regenerating the quinone structure, in a process that can be spontaneous or induced by oxidizing agents depending on the reaction conditions [131].

**Scheme 42:**
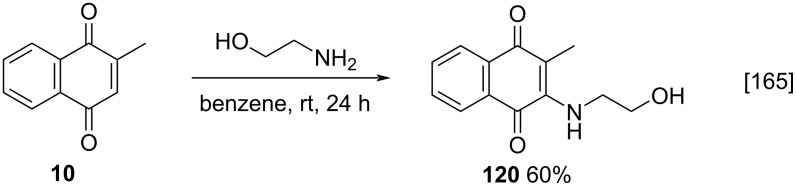
Michael-type addition of amines to menadione reported by Kallmayer.

The best-known method for the addition of nucleophiles to menadione was developed by Kallmayer [165]. In this method, initially proposed for the Michael-type addition of ethanolamine, menadione and an amine were solubilized in benzene and the reaction was maintained at room temperature (rt), leading to the amino-substituted menadione **120** in moderate yield (Scheme 42) [165]. Afterwards, ethanol/dichloromethane mixtures were used, as they increased the solubility of both menadione and the synthesized products.

**Scheme 43:**
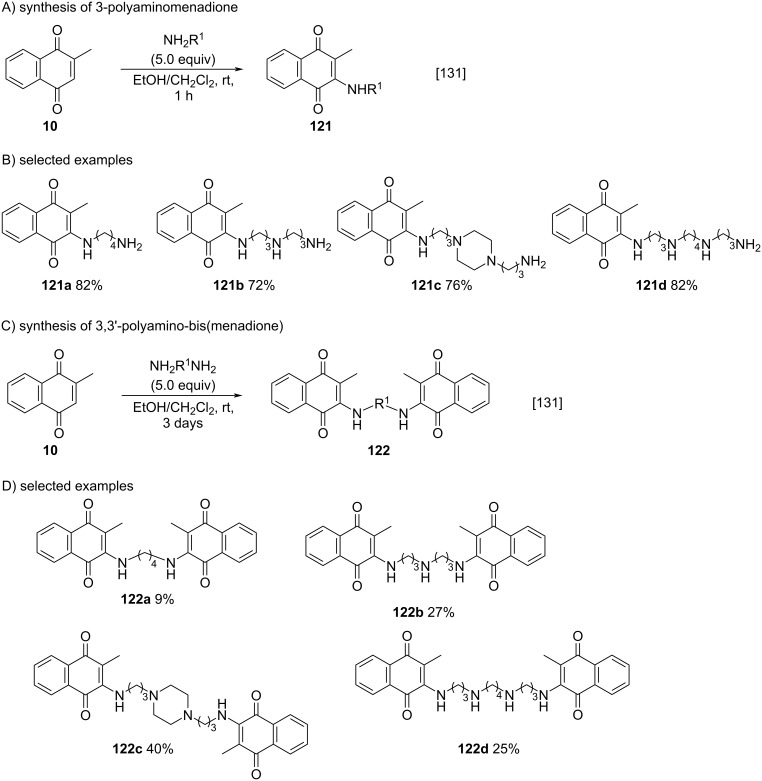
Synthesis of amino-menadione derivatives using polyalkylamines.

In recent years, the Kallmayer method [165] has been the most common approach to promote the addition of amines to menadione, proving to be quite efficient and robust [131,166–174]. Salmon-Chemin and co-workers described the synthesis of amino-substituted menadione derivatives using polyalkylamines to form the adducts 3-polyaminomenadione and 3,3'-polyamino-bis(menadione) [131]. Several reaction conditions were employed to obtain products **121a–d** or **122a–d**, such as the amount of polyamine and reaction time, requiring 5.0 equivalents of polyamine and 1 hour of reaction to form **121a–d** and 0.5 equivalents of polyamine and 3 days of reaction to obtain **122a–d**. The yields of each product type were also different, with **121a–d** being obtained in moderate to good yields and **122a–d** in low to moderate yields (Scheme 43).

**Scheme 44:**
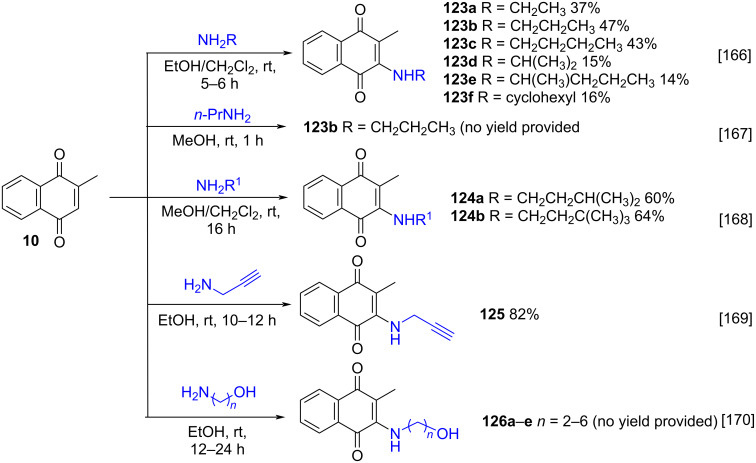
Selected examples for the synthesis of different amino-substituted menadione derivatives.

Karunan’s group [166], Wang’s group [167] and Li’s group [168] used the Kallmayer method to prepare aminomenadiones **123** and **124** through the addition of amines containing linear, cyclic, and branched aliphatic chains (Scheme 44). Jing and co-workers, in turn, applied this methodology for the addition of propargylamine to **10**, to form the propargylamino-substituted product **125** (Scheme 44) [169]. Bowen and co-workers also carried out the reaction between amino alcohols and **10**, in ethanol at rt, demonstrating that this method remains the most interesting option, even after 30 years, for the Michael-type addition of amino alcohols to **10**, yielding products of type **126** (Scheme 44) [170]. In all these cases, the yields of the adducts varied according to the nature of the respective precursor amine.

**Scheme 45:**
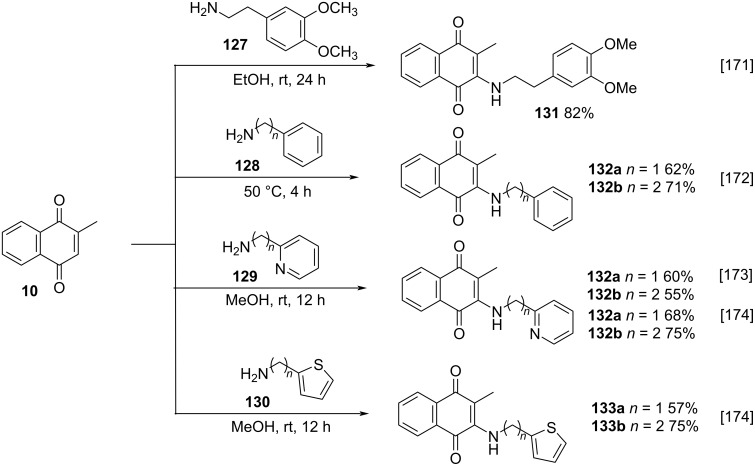
Selected examples of Michael-type addition of complex amines to menadione (**10**).

Other authors have also succeeded with the Michael-type addition of complex amines to menadione (**10**), containing arenes and heteroarenes as substituents in the aliphatic chain. In the work developed by Namsa-aid and Ruchirawat, homoveratrylamine (**127**) was used as nucleophile [171], while Zacconi and co-workers applied benzyl- or phenethylamines **128** [172]. However, the protocols required different solvents and reaction temperatures (Scheme 45). In the works developed by Wu and co-workers [173] and Patil and co-workers [174], amines containing heterocycles such as pyridine **129** or thiophene **130** were used as nucleophiles, to provide the corresponding compounds **132a**,**b** and **133a**,**b**, respectively, in good yields (Scheme 45). A possible reason for the higher yields obtained by Patil compared to those reported by Wu could have been an additional sonication step after the partial menadione dissolution in methanol, increasing the solubility of this reactant.

**Scheme 46:**
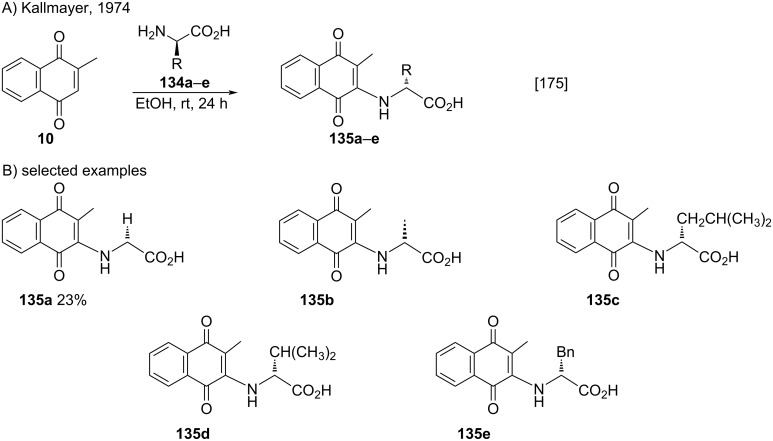
Addition of different natural α-amino acids to menadione.

The method developed by Kallmayer [165] also supports the use of amino acids, as described by Ge and co-workers [175]. They reported a Michael-type addition of different natural α-amino acids **134a–e** to menadione (**10**). However, the only product that formed with a measurable yield was the ʟ-glycine derivative **135a** (23% yield) (Scheme 46).

A variation of the Kallmayer method [165] was described by Mital and co-workers, which involved the addition of several amines to menadione in the presence of an inorganic base (K_2_CO_3_) to afford products **136** [176]. The compounds were obtained in moderate yields, in a very short reaction time, when compared to the original method (Table 6).

**Table 6:** Reaction conditions for the addition of amines to menadione by Mital and co-workers.

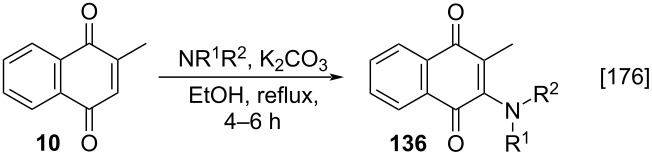			

Entry	Compounds	R^1^, R^2^	Yield (%)

1	**136a**	-H, -CH_2_Ph	45
2	**136b**	-H, -CH_2_CO_2_H	52
3	**136c**	-H, -CH_3_	45
4	**136d**	-H, -C(CH_2_)_5_	45
5	**136e**	-H, -Ph	44
6	**136f**	-CH_3_, -CH_2_Ph	39

Besides to the Kallmayer method [165], many other protocols for the Michael-type addition of amines to menadione have been developed and described. These protocols differ, basically, in the used solvent and the obtained adduct yields, when compared to the original method. With regard to solvents, the use of hot water [177], diethyl ether [178], acetonitrile at 45 °C [179], the amino reagent [180], and pure dichloromethane [181–183] were reported as solvents. Conditions that resulted in reduced menadione solubility, may explain the drop of the reaction yield.

**Scheme 47:**
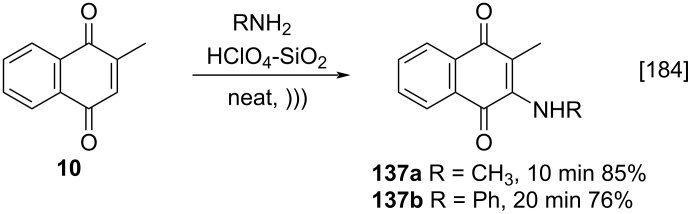
Michael-type addition of amines to menadione using silica-supported perchloric acid.

A particular method that deserves to be highlighted was described by Sharma and co-workers. It consisted in the addition of amines to menadione (**10**), via a solvent-free Michael-type addition, using silica-supported perchloric acid (HClO_4_-SiO_2_) and ultrasound irradiation (Scheme 47) [184] and provided the adducts **137a**,**b** in good yields in up to 20 minutes. This method is highly efficient and can be considered environmentally friendly when compared to the previously described protocols, which used solvents such as dichloromethane or required longer reaction times.

**Scheme 48:**
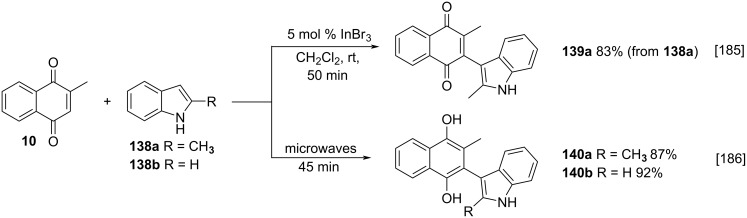
Indolylnaphthoquinone or indolylnaphthalene-1,4-diol synthesis reported by Yadav et al.

The Michael-type addition reaction of menadione could also be carried out using the appropriate indole to obtain indolylnaphthoquinones **139** or indolylnaphthalene-1,4-diols **140** in excellent yields. In this regard, Yadav and co-workers [185] reported the indium(III) bromide catalyzed conjugate addition of 2-methylindole (**138a**) to **10** to obtain the product **139a** (Scheme 48). The same group also published a microwave-accelerated solvent- and catalyst-free synthesis of 3-indolylhydroquinones **140a**,**b** (Scheme 48) [186].

**Scheme 49:**
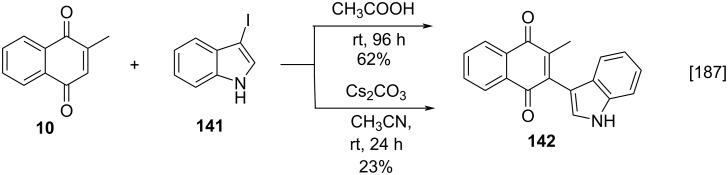
Indolylnaphthoquinone synthesis reported by Tanoue and co-workers.

Tanoue and co-workers [187] described the synthesis of indolylnaphthoquinone **142** using a Michael-type addition reaction of **10** with 3-iodoindole (**141**) (Scheme 49). The reaction was carried out in acetic acid at rt for 4 days resulting in 2-methyl-3-(3-indolyl)-1,4-naphthoquinone (**142**) in 62% yield. When the reaction was carried out in the presence of cesium carbonate in acetonitrile at rt for 1 day, the product **142** was obtained in 23% yield.

**Scheme 50:**
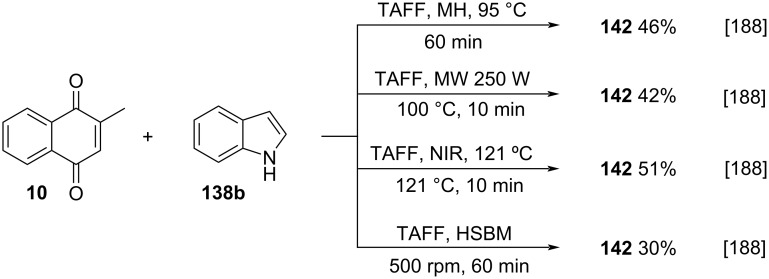
Indolylnaphthoquinone synthesis from menadione by Escobeto-González and co-workers.

An ecofriendly approach to this methodology was reported by Escobeto-González and co-workers [188], who investigated three different non-conventional reaction activation modes: microwave (MW) and near-infrared irradiation (NIR) as well as high-speed ball milling (HSBM) (Scheme 50). The alternative approaches were compared with typical mantle heating conditions (MH) and all methods were carried out under solvent-free conditions in the presence of Tonsil Actisil FF (TAFF) as a green catalyst. The best results were obtained using NIR at 121 °C for 10 min furnishing product **142** in 51% yield. According to the authors, the reaction mechanism proceeds via a classical Michael-type addition of indole (**138b**) to **10**, assisted by an oxygen interaction of a carbonyl group with the Lewis acidic sites of TAFF, followed by in situ oxidation to obtain the product **142** [188].

**Scheme 51:**
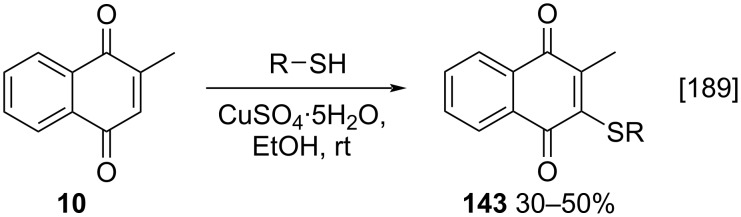
Synthesis of menadione analogues functionalized with thiols.

Thiols can also be employed as nucleophiles in the Michael-type addition to menadione. This approach is quite similar to the biological processes that naturally run between menadione and cysteine derivatives. Chen and co-workers described the synthesis of menadione analogues functionalized with thiols [189], using an adaptation of the already described method by Borovkov [190], where menadione was reacted with thioalcohols, thioethers, and ethers. The reaction occurs between menadione (**10**) and the respective thiols using copper sulfate pentahydrate as catalyst, in ethanol, at rt for 24 hours, to furnish products **143** in 30–50% yield (Scheme 51).

**Scheme 52:**
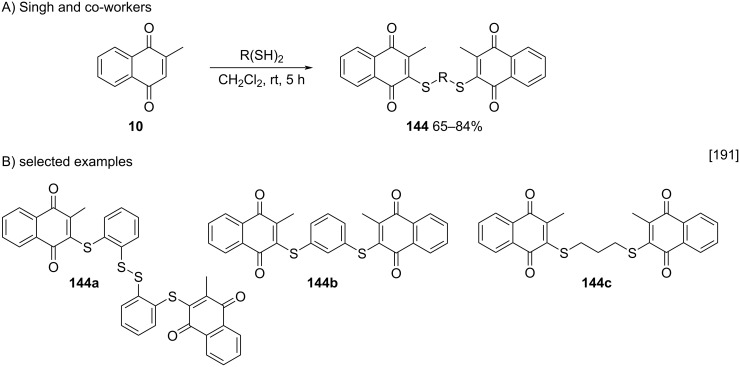
Synthesis of menadione-derived symmetrical derivatives through reaction with dithiols.

Singh and co-workers described the synthesis of bis-menadione derivatives through Michael-type addition of different dithiols and menadione used in excess [191]. The reaction proceeded in dichloromethane at rt for 5 h, furnishing products **144** in 65–84% yield. These results make this method very effective for the synthesis of menadione-derived symmetrical molecules (Scheme 52).

**Scheme 53:**
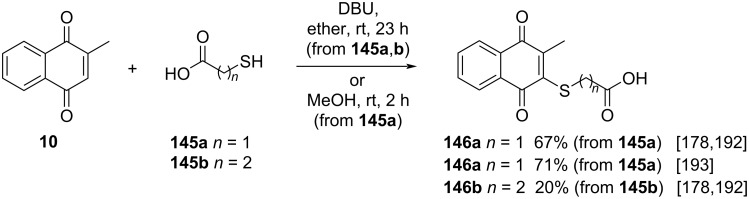
Mercaptoalkyl acids as nucleophiles in Michael-type addition reaction to menadione.

Mercaptoacetic (**145a**) and mercaptopropanoic (**145b**) acids were also used as nucleophiles in Michael-type addition reactions to menadione, as described by Garbay’s group [178,192] and Singh’s group [193]. In the method developed by Garbay and co-workers, that was based on Tamure and co-workers’ methodology for the addition of 2-mercaptoethanol to menadione (**10**) [194], the addition reaction occurred in the presence of DBU and ethyl ether, providing products **146a** and **146b** in 67% and 20% yield, respectively (Scheme 53). In turn, the method applied by Singh required milder conditions and was more effective, using only menadione (**10**), the nucleophile **145a** and methanol as a solvent to synthesize the addition product **146a** in 71% yield (Scheme 53).

**Scheme 54:**
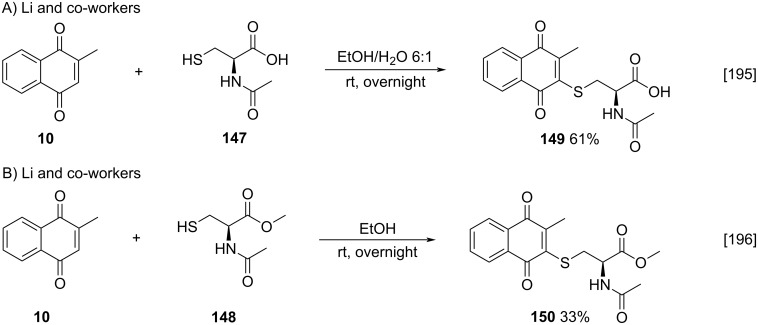
Reactions of menadione (**10**) with cysteine derivatives for the synthesis of quinoproteins.

In a study on the introduction of quinones into proteins to obtain quinoproteins, Li and co-workers reported a methodology for the reaction of menadione (**10**) with ʟ-cysteine [195]. The reaction of **10** and *N*-acetyl-ʟ-cysteine (**147**) occurs in an ethanol/water mixture, at room temperature overnight, to furnish the product **149** in 61% yield (Scheme 54A). Li and co-workers, in a similar study, reacted menadione (**10**) with *N*-acetylcysteine methyl ester (**148**) to obtain compound **150** in 33% yield [196] (Scheme 54B).

**Scheme 55:**
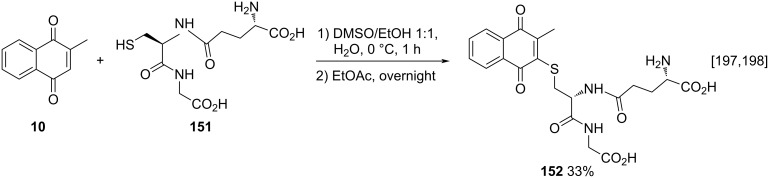
Synthesis of menadione-glutathione conjugate **152** by Michael-type addition.

Kumar and co-workers focused their work on the synthesis of menadione-glutathione conjugates by Michael-type addition reaction [197], based on the method of Nickerson and co-workers [198]. In this method an aqueous solution of ʟ-glutathione (**151**) was treated with a menadione (**10**) solution in DMSO/ethanol at 0 °C for 1 h, then diluted with ethyl acetate, and stirred at room temperature overnight (Scheme 55). The menadione-glutathione conjugate **152** was separated by filtration, being obtained in 33% yield without further purification.

## Conclusion

Organic synthesis is the most active subarea of chemistry that uses structural models to plan and develop new products and new reactions. The use of abundant natural products, even if produced by synthetic means, is one of the central strategies in research for the development of new bioactive compounds. Since the first reports on the biological activities of menadione and the development of methods for its preparation on an industrial scale, various compounds have been synthesized using this important 1,4-naphthoquinone. As a structural platform, this commercially available organic compound offers multiple possibilities for chemical modification in a search for new hit compounds that can become a new drug. This review represents an update and overview of aspects of menadione chemistry, synthetic opportunities, and its derivatives. The number of applications of menadione highlighted in this review clearly demonstrates the central role this compound plays in synthetic organic and medicinal chemistry.
